# An integrated analysis of molecular aberrations in NCI-60 cell lines

**DOI:** 10.1186/1471-2105-11-495

**Published:** 2010-10-06

**Authors:** Chen-Hsiang Yeang

**Affiliations:** 1 Institute of Statistical Science, Academia Sinica, Taipei, Taiwan

## Abstract

**Background:**

Cancer is a complex disease where various types of molecular aberrations drive the development and progression of malignancies. Large-scale screenings of multiple types of molecular aberrations (e.g., mutations, copy number variations, DNA methylations, gene expressions) become increasingly important in the prognosis and study of cancer. Consequently, a computational model integrating multiple types of information is essential for the analysis of the comprehensive data.

**Results:**

We propose an integrated modeling framework to identify the statistical and putative causal relations of various molecular aberrations and gene expressions in cancer. To reduce spurious associations among the massive number of probed features, we sequentially applied three layers of logistic regression models with increasing complexity and uncertainty regarding the possible mechanisms connecting molecular aberrations and gene expressions. Layer 1 models associate gene expressions with the molecular aberrations on the same loci. Layer 2 models associate expressions with the aberrations on different loci but have known mechanistic links. Layer 3 models associate expressions with nonlocal aberrations which have unknown mechanistic links. We applied the layered models to the integrated datasets of NCI-60 cancer cell lines and validated the results with large-scale statistical analysis. Furthermore, we discovered/reaffirmed the following prominent links: (1)Protein expressions are generally consistent with mRNA expressions. (2)Several gene expressions are modulated by composite local aberrations. For instance, CDKN2A expressions are repressed by either frame-shift mutations or DNA methylations. (3)Amplification of chromosome 6q in leukemia elevates the expression of MYB, and the downstream targets of MYB on other chromosomes are up-regulated accordingly. (4)Amplification of chromosome 3p and hypo-methylation of PAX3 together elevate MITF expression in melanoma, which up-regulates the downstream targets of MITF. (5)Mutations of TP53 are negatively associated with its direct target genes.

**Conclusions:**

The analysis results on NCI-60 data justify the utility of the layered models for the incoming flow of cancer genomic data. Experimental validations on selected prominent links and application of the layered modeling framework to other integrated datasets will be carried out subsequently.

## Background

Cancer is a systemic disease where alterations of various physiological processes drive the development and progression of malignancies (e.g., [1-5]). These alterations result from combinations of many cytogenetic/molecular aberrations such as large-scale karyotype changes (e.g., [6]), sequence alterations on protein-coding or regulatory regions (e.g., [7,9]), DNA copy number variations (e.g., [10]), epigenetic modification changes (e.g., [5,11]), alterations of mRNA (e.g., [12]), protein (e.g., [13]) and microRNA (e.g., [14]) expressions. A comprehensive characterization of a cancer system requires concurrent measurements of these diverse molecular aberrations in the same set of samples. Several international consortia and research institutions have launched large-scale projects to catalog the genomic, transcriptomic and epigenomic changes across multiple tumor types and generated preliminary data (e.g., [7,15,16]). In addition, comprehensive assays on the NCI-60 cancer cell lines have been performed by distinct research groups over the last two decades (e.g., [6,9,17,13,23]).

As the large-scale, comprehensive assays will be common in cancer research and prognosis, it is essential to perform integrative computational analysis of the heterogeneous data in order to obtain a systematic understanding of the underlying biology. Currently integrative analyses of cancer data focus on three interrelated directions. First, molecular biomarkers identified from each type of data were combined to improve the prognostic accuracy of tumors. Meta-analysis is typically applied to multiple datasets in tumor classification and prediction (e.g., [24-26]). Second, beyond single markers most recent studies examined the abnormal pathway activities by combining the molecular aberrations of their constituent genes (e.g., [12,15,16,27-29]). Third, some studies also tracked the causes of abnormal gene expressions by correlating them with DNA copy numbers, gene mutations, DNA methylations or microRNA expressions (e.g., [16,30-33]). Beyond cancer data various computational models of data integration have been applied to other datasets. Examples include probabilistic Bayesian models [34], probabilistic relational models [35], mutual information networks [36], module networks [37] and factor graphs ([38,39]).

Despite the rich literature of data integration in computational biology, several issues have not been widely addressed in cancer data analysis. First, most integrative cancer studies tend to apply case-by-case analysis to combine different types of data. For instance, a common method of integrating copy number and gene expression data is to calculate the correlation coefficients between DNA copy numbers and mRNA expressions of the same genes (e.g., [17,30]). This analysis only captures simple, pairwise relations of molecular aberrations and is difficult to extend to a wide variety of data. A coherent framework that unifies distinct types of molecular data in the same model is needed. While such probabilistic models have been applied to other organisms and the clinical data of human diseases, they have not been widely applied to the comprehensive molecular aberration data of cancer. Second, tracing the statistical and causal relations of multiple molecular aberrations is important for understanding the mechanisms of cancer phenotypes but is not emphasized in existing studies of cancer data integration. Pairwise correlations fail to distinguish between direct and indirect effects of molecular aberrations and cannot capture the combinatorial relations of multiple aberrations. For instance, the mRNA expression of a gene can be elevated by the amplification of its DNA copies or by the amplification of its transcription factor. A general statistical model that delineates the direct, indirect effects and combinatorial interactions is required. Third, genome-wide associations on heterogeneous data suffer from the curse of dimensionality as the number of probed features far exceeds the sample size. Various statistical techniques are proposed to alleviate the small-sample problem by controlling model complexity or assessing the reliability of multi-hypotheses tests (e.g., [40-42]). However, the association results are often difficult to interpret in terms of mechanisms. Some associations can be attributed to known molecular mechanisms (e.g., the frame-shift insertion/deletion of a gene disrupts its mRNA synthesis), while other associations may have unknown mechanistic links (e.g., a MAP kinase activates a gene expression via an unknown signaling pathway). These mechanistic information should be utilized to assign the confidence of associations and to identify the novel links not explained by known mechanisms.

In this study, we propose a *layered modeling framework *to tackle these issues. The goal of the model is to explain the molecular phenotypes - mRNA and protein expressions - with the observed molecular aberrations. Associations with phenotypes are classified into three layers according to the level of uncertainty and complexity for their mechanistic interpretations. Layer 1 models associate gene expressions with the molecular aberrations on the same loci. Layer 2 models associate the expressions with the aberrations on different loci but have known mechanistic links. Layer 3 models associate the expressions with nonlocal aberrations which have unknown mechanistic links. We sequentially incorporate the associations belonging to increasing layers to fit the phenotype data. Each layer of models are formulated by logistic regression models of multiple variables, and new variables that provide additional explanatory powers are included according to a nested hypothesis testing procedure. We applied the layered models to the integrated datasets of NCI-60 cell lines and discovered/reaffirmed prominent mechanistic links pertaining to major oncogenes and tumor suppressors including CDKN2A, TP53, MYB, MITF, PAX3, and other genes.

## Methods

### Overview of the integrated modeling framework

The goal of this work is to find the molecular aberrations that explain the variations of gene expressions across a panel of cancer and/or normal samples. We use logistic regressions to model the dependency of molecular aberrations and gene expressions for their simplicity and expressiveness for the effects of multiple inputs. Features (variables) are sequentially incorporated in the model based on statistical hypothesis tests. A new variable is added to an existing model if the joint model significantly outperforms both the existing model and the model with the new variable alone. To preserve the information of continuous data (e.g., mRNA expressions) we applied *probabilistic quantization *to convert intensities (e.g., log ratios in the mRNA expression data) into probabilities of each state (e.g., up/down regulation or no change). Probabilistic quantization, logistic regressions and model selection procedures are described in subsequent sections of *Methods *and Additional file 1 (Section *Data processing*).

**Figure 1:**
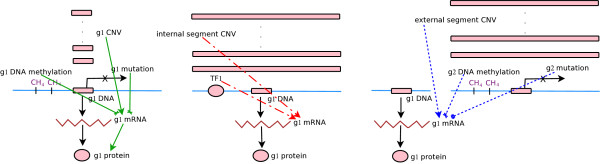
**Three-layered models connecting molecular aberrations and gene expressions**. Left: layer 1 models connecting mutations, CNVs, DNA methylations with mRNA and protein expressions of the same genes (*g*_1 _in the diagram). Solid black lines indicate transcription and translation. Solid green lines indicate layer 1 associations. Associations with mutations or DNA methylations are inhibitory. Middle: layer 2 models connecting internal segment CNVs and transcription factor expressions with expressions of another gene. Here *g*_1 _is a known target of transcription factor TF1. Dashed red lines indicate layer 2 associations. Right: layer 3 models connecting external segment CNVs, mutations and DNA methylations with mRNA expressions of a gene on another chromosomal segement. Dotted blue lines indicate layer 3 associations. Associations with external DNA methylations are inhibitory, whereas associations with external gene mutations can be positive or negative.

The novelty of our approach dwells on the priority of incorporating molecular aberrations in the model. We propose a *layered modeling framework *to identify the molecular aberrations explicating the expression data. The central idea is to sequentially apply a hierarchy of models with increasing complexity and uncertainty regarding the possible mechanisms connecting molecular aberrations and gene expressions. A more complex model is not incorporated unless either the aberration data required for simpler models are not available or the explanatory power of the former significantly surpasses the power of any simpler models. We classify the mechanistic models into three layers according to the directness and uncertainty connecting the causes (observed molecular aberrations) and effects (observed gene expressions). Figure 1 illustrates the relations of molecular aberrations in each class.

1. Layer 1 models: Local aberrations of a gene/genomic segment modulate the expressions/aberrations on the same locus. In this study the following layer1 models are considered: The karyotype of a chromosomal segment affects its copy number variation (CNV). The CNV, mutation and DNA methylation of a gene affect its expression. The mRNA expression of a gene modulates its protein expression.

2. Layer 2 models: Nonlocal aberrations modulate the expressions/aberrations on different loci, and the aberrations and phenotypes are connected via known mechanisms. In this study the following layer 2 models are considered: The CNV of a chromosomal segment affects the expression of a constituent gene on the segment whose CNV data is not available. The mRNA expression of a transcription factor affects the mRNA expressions of its known targets.

3. Layer 3 models: Nonlocal aberrations modulate the expressions/aberrations on different loci, and some links connecting the aberrations and phenotypes do not have known mechanistic correspondence. In this study the following layer 3 models are considered: The CNV of a chromosomal segment is associated with the expression of a gene on another chromosomal segment. The mutation or DNA methylation of a gene is associated with the expression of another gene.

To explain the expression data of a gene we incrementally incorporate observed aberrations from layer 1 to layer 3 models. Local aberrations on the same locus are considered first because they can provide a direct mechanistic explanation for expression changes. Nonlocal aberrations with known mechanistic links to expressions are then introduced since they are consistent with the known mechanistic models and require few additional assumptions to fit the data. Indirect associations with chromosomal segment CNVs, gene mutations, and DNA methylations are invoked only when the layer 3 models significantly outperform the lower-layered models.

### Data sources and processing

Seven datasets on NCI-60 cell lines were downloaded from the website of the Genomics and Bioinformatics Group at NCI [8]: mutation analysis of 24 cancer genes [9], Comparative Genomic Hybridization (CGH) array data of DNA copy number variations [17], spectral karyotyping data [6], cytosine methylation profiling on promoters [18], cDNA microarray data [21] and Affymetrix transcript profile data [22] of mRNA expressions, and protein expression profiles [13]. The union of these datasets probed 14856 genes.

The known targets of 643 human transcription factors were extracted from the TRANSFAC database [43]. Functional descriptions and chromosome coordinates of 39942 human genes were downloaded from the NCBI database [44]. 4291 cancer-related genes were extracted from the OMIM database [45].

To utilize the datasets from diverse sources we adopted the following normalization procedures. Mutation states were treated as binary random variables and all other aberrations/responses were treated as discrete random variables with three possible states - up-regulation, down-regulation and no change. We applied *probabilistic quantization *to convert the measurement values into the probabilities of the three possible states. Briefly, denote *z_ij _*the observed value of gene *i *on cell line *j*, and *x_ij _*its discrete hidden state. *z_ij _*was first rank-transformed into the cumulative distribution function (CDF) value *y_ij_*. Families of polynomial quantization functions $f_{\gamma }(y_{ij})\equiv y_{ij}^{\gamma }$ and ${\bar{f}}_{\gamma }(y_{ij})\equiv {\left(1-y_{ij}\right)}^{\gamma }$ then converted *y_ij _*into the probabilities of the hidden state *P*(*x_ij _*= 1|*y_ij _*, *γ *) and *P*(*x_ij _*= -1|*y_ij_*, *γ *). Finally *P*(*x_ij _*|*y_ij_*) was obtained by integrating over the parameter *γ *of quantization functions (Additional file 1, Figure S1). Probabilistic quantization preserves the information of continuous data and makes it accessible for simple discrete models such as logistic regressions and Bayesian networks. Details of the probabilistic quantization procedures are described in Additional file 1 (Section *Data processing*).

The cDNA and Affymetrix expression data intersect in 5251 genes. Intra-gene correlation coefficients are significantly higher than the background distribution of all probes between the two datasets (Additional file 1, Figure S2; the means of the intra-gene correlation coefficients and the background distribution are 0.4 and 0 respectively, the Komolgorov-Smirnov p-value < 10^-100^), confirming the consistency of cDNA and Affymetrix data. We then combined the two datasets by selecting the genes which either had consistent cDNA and Affymetrix profiles or were probed in only one dataset. Overall, the combined dataset contains 8094 gene expressions (4231 cDNA and 3863 Affymetrix data). Details of the dataset combination procedures are described in Additional file 1 (Section *Combination of cDNA and Affymetrix data*).

### Logistic regression models of molecular aberrations

We used logistic regressions to model the effects of molecular aberrations on gene expressions. Denote *y *the expression of a gene and ***x ***the local and nonlocal aberrations that affect *y*. The conditional probability is

(1) $$P(y|x)=\frac{1}{Z(x)}e^{\Sigma_{i\;}\lambda_{i}\;f_{i}(x)y},\;\lambda_{i}\geq 0,\;\forall i.$$

*f_i_*(***x***)'s are scalar feature functions specifying the relations of ***x ***and *y*. *λ_i_*'s are nonnegative parameters, and *Z*(***x***) is the partition function that normalizes the conditional probabilities. In this work, *f_i_*(***x***)'s are determined by explicit assumptions about the effects of molecular aberrations on gene expressions. *f*(*x*) = *x *if aberration *x *activates expression *y*. *f*(*x*) = -*x *if aberration *x *represses expression *y*. In our study only two types of relations are repressive: the effects of DNA methylations on gene (mRNA or protein) expressions and the effects of mutations on the negatively associated gene expressions.

A model contains multiple feature functions if the combinations of multiple aberrations explain the data. For instance, if gene expression *y *is repressed by either mutation *x*_1 _or DNA methylation *x*_2 _of the same gene, then the exponent of equation 1 is -*λ*_1_*x*_1 _- *λ*_2_*x*_2_.

The log likelihood of a logistic regression model has the following form:

(2) $$\begin{array}{l} L(x,\;y)=\sum_{k=1}^{n}\log(P(x_{k}))+\log(\frac{1}{Z(x_{k})})+\sum_{i}\lambda_{i}f_{i}(x_{k})y_{k}\\ \;\;\;\;\;\;\;\;\;\;\;\;\;\;\;\;\;=\sum_{C_{x},C_{y}}N(C_{x})\log(P(C_{x}))+\\ \;\;\;\;\;\;\;\;\;\;\;\;\;\;\;\;\;\;\;\;\;N(C_{x},\;C_{y})[-\log(Z(C_{x}))+\sum_{i}\lambda_{i}f_{i}(C_{x})C_{y}]. \end{array}$$

where *C**_x _***and *C_y _*stand for configurations of ***x ***and *y*, and *N*(*C***_*x*_**), *N*(*C***_*x*_**, *C_y_*) the frequencies of their occurrences. Probabilities of input variables *P*(***x***_*k*_) are modeled as independent multinomial distributions with a uniform Dirichlet prior. Probabilistic quantization yields *fractional counts *on each configuration for each sample. On a specific cell line *i *the fractional counts of *y_i _*= -1, 0, 1 are *P*(*y_i _*= -1), *P*(*y_i _*= 0), *P*(*y_i _*= 1) obtained from probabilistic quantization. *N*(*C**_x_***) and *N*(*C**_x_***, *C_y_*) are calculated by summing the fractional counts of each configuration over the samples. The maximum likelihood parameters of *λ_i_*'s are numerically estimated using the Newton-Raphson method.

### Inferring segment CNVs from CGH data

The CGH array data from [17] provides a sparse sampling on the genome-wide CNVs as only 219 genes contain valid CGH data. However, since events of copy number changes often cover multiple adjacent loci on a chromosome, it is possible to extrapolate the CGH data of sparse probes into the CNVs of consecutive chromosomal segments. Spatial dependency of CNVs is manifested from the measurements of 219 genes on a CGH array [17] and confirmed in Additional file 1, Figure S3. Most strongly correlated pairs appear in the adjacent probes of the same chromosomes. Comparison of the distributions of correlation coefficients of adjacent genes versus the entire gene set also supports the spatial dependency (Komolgorov-Smirnov p-value < 8.44 × 10^-296^).

However, not all the genes on the same chromosomes are highly correlated. Some chromosomes can be partitioned into multiple segments where intra-segment genes are highly correlated and inter-segment genes are poorly correlated. We devised a recursive algorithm to partition each chromosome into correlated segments. In brief, the CGH data of all probes on the same segment were treated as instantiations of a common hidden variable (a naive Bayes model, [46]). The algorithm iteratively partitions a segment that optimizes the joint likelihood of the naive Bayes model and stops when further partitions do not improve the likelihood score. Detailed procedures of the partitioning algorithm are described in Additional file 1 (Section *Partitioning a chromosome into segments according to CGH data*).

Using this algorithm we partitioned 23 chromosomes (there are no probes on the Y chromosome) into 49 segments. Table 1 reports the chromosome coordinates, the numbers of NCBI genes and CGH probes on each segment. The correlation coefficients of intra-segmental probes are substantially higher than those of inter-segmental probes (Additional file 1, Figure S4; mean correlation coefficients 0.65 and 0 respectively, Komolgorov-Smirnov p-value < 10^-260^). Disparity between the two distributions justifies the accuracy of the partitioning algorithm.

**Table 1:** Location and functional information of chromosome segments. *N*_1_: # associated intra-segmented genes, *N*_2_: # associated inter-segment genes.

index	location	length(Mb)	# genes	# CGH probes	amplified tissues	*N*_1_	*N*_2_
1	chr1p 31.00-36.20	49.39	549	7	CNS leukemia	143	28
2	chr1p 13.00-33.00	47.14	253	6	CNS	59	79
3	chr1q 21.00-23.00	6.97	282	2	-	0	0
4	chr1q 23.00-43.00	76.99	419	8	CNS lung	76	20
5	chr2p 12.00-24.10	59.28	406	7	melanoma lung	82	36
6	chr2q 23.00-24.00	7.39	542	2	-	23	11
7	chr3p 24.20-25.00	11.83	118	2	-	18	19
8	chr3p 14.30-21.30	4.08	235	4	-	50	59
9	chr3p 13.00-14.20	11.29	71	2	melanoma	11	130
10	chr3q 13.30-28.00	73.57	375	11	-	81	78
11	chr4p 16.30-16.30	1.17	182	2	-	29	119
12	chr4q 21.00-31.21	56.74	256	6	-	0	0
13	chr4q 31.00-35.10	39.39	110	2	-	0	0
14	chr5q 35.10-35.10	1.64	130	2	lung ovary	20	18
15	chr5q 31.00-31.10	4.22	514	2	-	38	9
16	chr6p 21.20-21.30	11.05	554	3	-	87	49
17	chr6q 21.00-27.00	58.79	278	5	leukemia	30	38
18	chr7q 21.00-22.00	4.07	318	3	-	16	19
19	chr7q 21.00-31.00	23.80	347	3	-	40	19
20	chr8p 11.00-22.00	30.75	182	3	-	36	10
21	chr8q 11.00-13.00	0.23	305	2	-	0	0
22	chr9q 22.30-22.30	0.19	312	2	-	13	26
23	chr9q 34.00-34.10	4.22	236	4	-	34	49
24	chr10q 11.20-26.00	80.46	527	4	-	86	95
25	chr11p 15.40-15.50	3.29	84	3	-	17	23
26	chr11p 13.00-15.50	27.17	165	2	-	34	14
27	chr11q 11.00-13.00	2.33	181	2	-	0	0
28	chr11q 21.00-23.20	18.98	213	5	ovary	44	18
29	chr11q 13.00-14.00	0.80	81	2	-	11	47
30	chr11q 13.00-13.00	0.76	141	3	-	2	17
31	chr12q 13.00-13.30	8.77	479	3	-	41	49
32	chr12q 14.30-24.30	35.40	317	4	-	68	27
33	chr13q 12.30-21.20	27.85	253	4	colon	52	49
34	chr14q 11.20-32.32	84.34	470	5	-	150	46
35	chr15q 22.00-26.10	23.92	441	6	-	94	31
36	chr16p 13.10-13.30	13.53	342	2	-	40	35
37	chr16q 22.10-24.30	20.77	285	2	-	41	29
38	chr17p 13.00-13.30	6.57	245	3	-	47	46
39	chr17q 11.20-22.00	3.42	336	4	-	85	24
40	chr17q 22.00-23.20	4.63	284	3	-	32	28
41	chr18p 11.20-11.31	8.89	213	3	-	17	31
42	chr19q 12.00-13.40	21.22	947	5	-	82	15
43	chr20p 13.00-13.00	2.40	75	3	-	23	14
44	chr20p 11.23-13.00	0.71	68	2	-	11	26
45	chr20q 13.00-13.31	10.16	92	7	-	8	6
46	chr20q 11.20-13.20	13.89	160	11	-	9	5
47	chr21q 22.10-22.30	13.33	154	6	leukemia	41	14
48	chr22q 12.20-13.10	9.92	376	3	leukemia melanoma	48	81
49	chr23q 11.20-28.00	80.71	605	2	-	76	12

The CGH data of the probes on the same chromosomal segments were combined to infer the CDF values of the segment CNV data. We again treated the segment CNV as a hidden variable of a naive Bayes model and the CGH data of probes on the segment as instantiations of the hidden variable. For each sample *i*, the log likelihood of the model is

(3) $$L(x_{i},\;y_{i})=\log P(x_{i})+\sum_{j}\log P(y_{ij}|x_{i}).$$

where *x_i _*is the hidden segment CNV on sample *i *and ***y****_i _*the observed CGH data on sample *i*. ***y****_i _*is indexed by probe *j *on the segment. By substituting *x_i _*= 0, ± 1 into equation 3 we obtain the unnormalized posterior probability *P*(*x_i_*|***y****_i_*):

(4) $$P(x_{i}=x|y_{i})\propto e^{L(x_{i}=x,y_{i})}.$$

The CDF value of *x_i _*is obtained by applying the inverse function of probabilistic quantization to *P*(*x_i _*= *x*|***y****_i_*).

### Model selection

To identify the molecular aberrations explaining a gene expression data, we incrementally augmented the model by launching a series of hypothesis tests. A new variable is included in the model if the augmented model significantly outperforms the old model in fitting the data. Molecular aberrations are prioritized according to the layered model classifications. The following model selection procedures were applied to each gene expression data.

1. Initially set the null model *M*_1 _= *M*_0 _to be independent; i.e., no aberration explains the data. Evaluate the log likelihood ${\hat{L}}_{0}$ of the independent model using a multinomial distribution.

2. Iteratively execute the model augmentation function *augment*(*M*_1_):

(a) Select an aberration variable *x *according to the layered modeling framework priority. Create *M*_2 _⊃ *M*_1 _by augmenting *M*_1 _with a link connecting *x *to the expression data *y*. Also create ${M^{'}}_{1}⊃M_{0}$ by adding the (*x*, *y*) link to the independent model *M*_0_. *M*_2 _is the joint model of using *M*_1 _and *x *together to explain *y*. ${M^{'}}_{1}$ is the model of using *x *alone to fit *y*.

(b) Estimate the maximum likelihood parameters of the logistic regression models of *M*_1_, ${M^{'}}_{1}$ and *M*_2_.

(c) Substitute the maximum likelihood parameters into equation 2 to calculate the log likelihood values ${\hat{L}}_{1}$, ${{\hat{L}}^{'}}_{1}$ and ${\hat{L}}_{2}$.

(d) Perform three hypothesis tests:

(5) $$R_{21}=2\frac{{\hat{L}}_{2}}{{\hat{L}}_{1}},\;R_{21^{'}}=2\frac{{\hat{L}}_{2}}{{{\hat{L}}^{'}}_{1}},\;R_{1^{'}0}=2\frac{{{\hat{L}}^{'}}_{1}}{{\hat{L}}_{0}}.$$

The p-value of a log likelihood ratio is the maximum of the p-values obtained by both *χ*^2 ^approximation and random permutations of the input vectors of the models. Calculate the p-values *p*_21_, *p*_21*' *_and *p*_1*'0 *_for the three tests.

(e) Perform model selection according to *p*_21_, *p*_21*' *_, *p*_1*'*0 _and a pre-specified threshold *θ*. The following cases are considered:

- Both *p*_21 _and *p*_21*' *_≤ *θ *: *M*_1 _← *M*_2_.

- *p*_21 _≤ *θ*, *p*_21*' *_>*θ*, and *p*_1*'*0 _≤ *θ*: $M_{1}\leftarrow {M^{'}}_{1}$.

- *p*_21 _>*θ*, *p*_21' _>*θ*, and *p*_1'0 _≤ *θ*: $M_{1}\leftarrow M_{1}\cup {M^{'}}_{1}$.

- Otherwise: *M*_1 _← *M*_1_.

In case 1, the joint model *M*_2 _is significantly better than the current model *M*_1 _and the additional variable ${M^{'}}_{1}$, so *M*_2 _replaces *M*_1_. In case 2, the explanatory power of the additional variable ${M^{'}}_{1}$ covers that of the current model *M*_1_, so ${M^{'}}_{1}$*replaces M*_1_. In case 3, both *M*_1 _and ${M^{'}}_{1}$ significantly fit the data but the joint model *M*_2 _does not outperform neither of them. So both *M*_1 _and ${M^{'}}_{1}$*coexist *as valid explanatory models. The explanatory power of ${M^{'}}_{1}$ is covered by *M*_1 _in other cases, hence the additional variable is discarded.

(f) Iteratively incur *augment *(*M*_1_) if there are aberration variables remained.

Like other greedy algorithms the inferred associations depend on the order of including the features in the analysis. Despite this drawback the following properties of the association problem justify the feature inclusion orders of the algorithm. First, the orders of feature additions are prioritized by their mechanistic information. Candidates with local or non-local mechanistic evidence (layer 1 and 2 associations) are preferred to candidates without mechanistic evidence (layer 3 associations). Second, within each layer the ordering of including features does not follow an explicit rule. However, since the model selection algorithm can retract a model with inferior explanatory power and allow multiple coexisting models, the artifacts generated from a specific model selection order should be minimized.

### Other computational procedures

Detailed procedures of carrying out simulation studies, identification of putative regulators, identification of the putative targets of TP53 mutations, clustering the DNA methylation data, estimation of the false discovery rates, evaluation of functional enrichment of the inferred modules are all reported in Additional file 1.

## Results

### Simulation analysis

The primary merits of the layered models using logistic regressions are reduction of possible confounding factors of indirect associations and capacity in capturing the effects of multiple inputs. To justify these advantages we generated simulated data of varying gene numbers and noise levels and compared the inferred layered models with the results of two additional methods. Each gene expression was modulated by one or two of the following aberrations: local mutation and DNA methylation, CNV of its chromosome, and the expression of a master transcription factor on another chromosome (its targets were not given when learning the models). Nine combinations of gene numbers (10, 50 and 100 genes) and noise levels (additive, zero-mean Gaussian noise with standard deviations 0.2, 0.3 and 1.0) were used to generate the simulated data. Two other methods were applied to learn the simulated data: the Bayes Net Toolbox for Bayesian network structure learning [47] and k-means clustering on Matlab (k = 4 where accuracy was optimal). Each of these methods obtained a directed graph specifying the statistical relations of the aberrations. Accuracy was determined by counting overlapped edges of the networks of the underlying models and the graphs derived from the inferred models. We also applied the module network learning algorithm [48] to the simulated data of 10 genes but did not show them since the sensitivity was considerably smaller than the other three methods. The procedures of data simulation and model comparison are described in Additional file 1 (Section *Simulation studies*).

**Figure 2:**
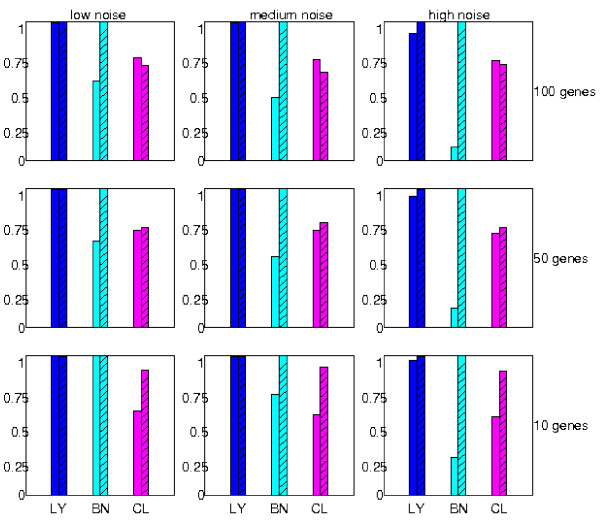
**Comparison of prediction accuracy on simulated data**. Sub-figures demonstrate the inference results with varying noise levels (horizontal direction, additive zero-mean Gaussian noise with standard deviations 0.2, 0.3 and 1.0) and gene numbers (vertical direction, 10, 50 and 100 genes). Blank colored bars denote the mean sensitivity and striped colored bars denote the mean specificity over 10 experiments in each setup. Blue bars (LY) denote layered models. Cyan bars (BN) denote Bayesian network learning. Magenta bars (CL) denote k-means clustering with *k *= 4.

Figure 2 shows the sensitivity and specificity of the three methods on the datasets with varying gene numbers and noise levels. The layered models (blue bars) achieve near 100% sensitivity and specificity in each experimental setting. Bayesian networks (cyan bars) have comparable sensitivity and specificity at low noise levels and gene numbers. However, sensitivity drops substantially with respect to increasing noise levels and moderately with respect to increasing gene numbers. For instance, sensitivity is 27% in the datasets of 10 genes and high noise level and 58% in the datasets of 100 genes and low noise level. The smaller number of true positives are likely due to a conservative model averaging procedure in learning the network structure. K-means clusters (magenta bars) have stable yet inferior sensitivity (between 60% and 70%) and specificity (between 70% and 90%) at each noise level and gene number setting.

### Summary of the inference results

We applied the layered modeling framework to seven datasets of NCI-60 cell lines, including mutational states, mRNA and protein expressions, copy number variations, cytosine methylations, and spectral karyotypes. Table 2 shows the number of gene expressions explained by each type of aberration in each layer. Thresholds of log-likelihood ratios and p-values for model selection were 3.0 and 0.1. Overall, 4364 of 8094 mRNA/protein expressions are associated with layered models. Both internal segment CNVs (segment CNVs associated with genes on the same segments) and external segment CNVs (segment CNVs associated with genes on different segments) explain the highest number of gene expressions (2165 and 1547 genes respectively). DNA methylations and mutations of some cancer-related genes are also associated with many gene expressions (935 and 392 respectively). In contrast, only a small fraction of gene expressions are explained by local aberrations or the expressions of their known transcription factors.

**Table 2:** Summary of gene expression data explained by each type of aberration.

layer	aberration	# explained genes validation	
1	internal CNV	37	coverage 31.9% (37 of 116)
1	internal mutation	3	coverage 50% (3 of 6)
1	internal methylation	15	coverage 7.98% (15 of 188)
1	internal mRNA	46	coverage 68% (46 of 68)
2	internal segment CNV	2165	coverage 26.77% (2165 of 8067)
2	transcription factor	6	coverage 3.87% (6 of 155)
3	external segment CNV	1547	3 TFs examined, 2 verified
3	external mutation	392	1 TF examined and verified
3	external methylation	935	7 TFs examined, 1 verified

False discovery rates (FDR, [40]) were evaluated for the associations of each layer and for all the associations. We adopted the permutation tests described in [42] as the null model and calculated two types of FDRs: (1) $\frac{\text{expected }\#\text{ false positives according to the null model}}{\#\text{ positive calls from the data}}$, (2) $\frac{\#\text{ false positives in the 99 percentile of the null model}}{\#\text{ positive calls from the data}}$

. Detailed description about the FDR calculations is reported in Additional file 1, Section *Estimation of false discovery rates*. The FDR of the first type among all association links is 20.34%, and the FDRs among the links of layer 1, 2 and 3 models are 6.65%, 7.82% and 27.39% respectively. The FDR of the second type among all association links is 27.29%, and the FDRs among the links of layer 1, 2 and 3 models are 11.39%, 15.13% and 38.11% respectively. The increasing FDRs over the layers are sensible, as layer 1 and 2 models are constrained by known mechanistic links and are thus less likely to be spurious. Furthermore, the FDRs calculated from the expected number of false positives are lower than those calculated from the 99 percentile of the null distributions. This is also sensible since the latter gives a much more conservative estimate of the false positive numbers. In both cases the high FDRs for layer 3 associations indicate the need for additional evidence to verify these links.

Quantities pertaining to the accuracy of the association outcomes - false positive and false negative rates from the empirical data - are much more difficult to acquire due to incomplete and sporadic knowledge about the relations of gene aberrations. We adopt the following procedures to gauge the accuracy of the associations. All the layer 1 and layer 2 associations possess mechanistic justifications because layer 1 and layer 2 models are based on known or plausible mechanisms of gene regulation. However, plausible associations are not necessarily manifested as they might be interfered by additional mechanisms. For instance, a protein expression may not be correlated with its mRNA expression due to post-transcriptional regulation. For layer 1 and layer 2 models, we reported the coverage of significant associations relative to the possible mechanistic links in Table 2. A high percentage of mRNA-protein expression pairs possess significant associations (46 of 68). In addition, a relatively high proportion of gene expressions are associated with the CNVs on the same loci or chromosomal segments (37 of 116 and 2165 of 8067 respectively). In contrast, the coverage of other known mechanisms is low. For instance, 155 genes are the known targets of valid transcription factors (genes with valid expression data), but only 6 of them are associated with their transcription factor expressions. In addition, we compared the layer 3 associations of transcription factors with their putative targets from literature and reported enrichment outcomes.

To validate the accuracy of layer 3 associations we solicited the top-ranking associations according to their likelihood scores and attempted to find supporting evidence from previous studies. We categorize the results of literature search into 3 classes: (1)Previous studies provide direct evidence for the associations. For instance, knock-out experiments or ChIP-seq assays identified the targets of a transcription factor, (2)Previous studies contain indirect evidence for possible associations. For instance, two associated genes are co-expressed in specific tissues of non-NCI-60 datasets, (3)There is no evidence of associations from pubmed keyword search. False positives - associations which are known to be false - are difficult to obtain since negative results are often not reported.

Additional file 1, Table S1 shows the literature search results for top associations of layer 3 models. 10 of top 50 associations with external segment CNVs possess indirect evidence. The genes are either on the same chromosomes of the segments or are putatively regulated by the genes on the segment. 5 of top 50 associations with external gene mutations possess direct evidence, and 16 associations possess indirect evidence. 13 of top 50 associations with external DNA methylations possess indirect evidence. Biological implications of several top-ranking associations are elaborated in the subsequent sections, and detailed description about literature review is reported in Additional file 1 (Section *Global accuracy validation of inferred associations*).

In addition to literature survey we also examined the functional enrichment of the gene groups associated with each aberration. About half of the gene groups are enriched with some GO classes or annotated pathways. The results are reported in Additional file 1, Table S2 (Section *Functional enrichment analysis*).

### Expression data explained by known mechanisms

#### Associations with local aberrations

2239 gene expressions are explained by local or non-local aberrations with known mechanisms. 56 mRNA and 47 protein expressions are explained by local aberrations of the same genes. Overall, a small fraction of gene expressions are explained by layer 1 models. Valid mutation, CNV and DNA methylation data (for each gene, less than 5 cell lines have missing values) cover only 24, 219 and 320 genes respectively. By restricting to the genes where any of these local aberrations is probed, 20% of mRNA expressions (56 of 280) are explained. The low number suggests many mRNA expressions are modulated by aberrations on other loci. In contrast, 68% of valid protein expressions (46 of 68) are positively associated with the mRNA expressions of the same genes. Consistency of mRNA and protein expression data is prominent when comparing the intra-gene correlation coefficients with the all-versus-all correlation coefficients between the two datasets (Additional file 1, Figure S5; Komolgorov-Smirnov p-value < 1.26 × 10^-27^). The results corroborate the quality of mRNA and protein expression data.

**Figure 3:**
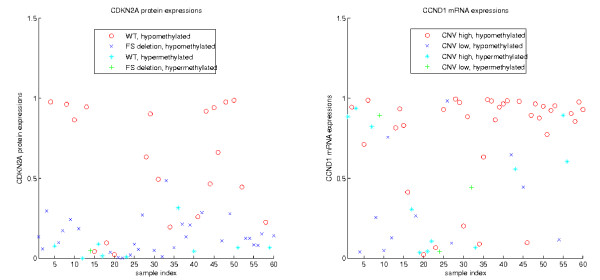
**Two gene expressions explained by composite aberrations**. Left: CDKN2A protein expressions are repressed by either frame-shift deletions or DNA methylation. Right: CCND1 mRNA expressions are activated by high copy numbers and repressed by DNA methylation. WT: wild type, FS: frame shift mutation, CNV: copy number variation.

Some gene expressions are explained by the models with composite (multiple) aberrations. In this case the expression level of a gene depends on the combination of multiple local aberrations. Two examples of composite models are shown in Figure 3. The protein expression of the tumor suppressor CDKN2A (TP16) is repressed by either frame-shift mutations or DNA methylations of the gene. Cell lines carrying either one of these aberrations have low expression levels, whereas cell lines without these aberrations tend to have high expression values. Similarly, the mRNA expression of the oncogene CCND1 (cyclin D1) is associated with both CNV and DNA methylation. Samples with high copy numbers and without DNA methylation have overall high expression levels, whereas samples with either low copy numbers or DNA methylation tend to be down-regulated.

Previous studies indicate that DNA copy numbers of some genes modulate their expression levels [10]. Positive associations with CNVs constitute the bulk of layer 1 models explaining mRNA expressions. Among the 116 genes where both CNVs and mRNA expressions are probed, 37 mRNA expressions are associated with the local CNVs (coverage rate 31.9%). Top genes with strong CNV-mRNA associations include NRAS (log likelihood ratio 12.74, p-value < 10^-4^), APEX (log likelihood ratio 12.36, p-value < 3 × 10^-4^), NFKB2 (log likelihood ratio 12.32, p-value < 0.0012), RAF1 (log likelihood ratio 12.17, p-value < 10^-6^), and CCNA2 (log likelihood ratio 11.67, p-value < 0.002). The strong associations of RAF1, APEX and CCNA2 were reported in a comparative analysis of the same datasets [17].

DNA methylation on promoters alters the chromatin structure surrounding the transcription start sites and silences gene expressions ([5,11]). In NCI-60 fewer mRNA expressions are negatively associated with DNA methylations on their promoters. Among the 188 genes where both DNA methylations and mRNA expressions are probed, only 15 have significant associations (coverage rate 7.98%). Two top genes with strong methylation-expression associations are PAX8 (log likelihood ratio 11.28, p-value < 0.0025) and SMO (log likelihood ratio 10.56, p-value < 0.001).

Mutations on a gene may enhance, repress or have no effect on its function [49]. However, frame-shift insertions/deletions will disrupt reading frames and reduce expressions. Among the 6 genes where mutations occur in at least 10 cell lines, we found strong negative associations of frame-shift mutations and expressions on tumor suppressors TP53 (log likelihood ratio 8.28, p-value < 0.05), CDKN2A (log likelihood ratio 5.43, p-value < 10^-3^) and PTEN (log likelihood ratio 10.75, p-value < 3 × 10^-4^). The coverage rate is 50%. In contrast, there is no apparent association of point mutations and gene expressions.

#### Cis-regulatory associations with segment CNVs

Most expressions are not associated with local aberrations of the same genes due to the sparseness of the aberration data and possible *trans-regulation *of genes on other loci. Layer 2 models associate non-local aberrations and gene expressions with known mechanistic links. In this study two types of layer 2 associations are considered: associations of chromosomal segment CNVs with the expressions of their constituent genes, and associations of the expressions of transcription factors and their known targets. Overall, internal segment CNVs explain a large number of gene expressions, whereas only a few associations of transcription factors with their known targets are found.

The expressions of 2165 genes are associated with their segment CNVs (coverage rate 26.77%). Table 1 reports the number of intra-segment genes associated with each segment CNV. The number of genes explained by internal segment CNVs is evidently proportional to the gene density and length of the segments (correlation coefficients 0.66 and 0.58 respectively).

In NCI-60 11 chromosomal segments have tissue-specific CNV profiles (Table 1 and Additional file 1, Figure S6). These segment CNVs are associated with many tissue-specific expressions of their constituent genes, suggesting that some tissue-specific expressions may arise from the copy number changes of the chromosomal segments. For instance, segment 2 (chromosome 1p 13-33) is amplified in the Central Nervous System (CNS) cell lines, and the associated genes are enriched in the GO category of neural development. Segment 9 (chromosome 3p 13-14.20) is amplified in melanoma, and the associated genes are enriched in the GO category of transporter activity.

Tumor phenotypes arising from the copy number changes of oncogenes or tumor suppressors are reported [10]. We extracted 4291 cancer-related genes from the OMIM database (OMIM) and identified 452 cancer-related genes associated with the CNVs on their chromosome segments. The results are reported in Addition file 1, Table S3.

#### Trans-regulatory associations with transcription factor expressions

Besides the *cis-regulation *of segment CNVs on their constituent gene expressions, transcription factors may regulate their targets on other chromosomes. To identify these *trans-regulatory *relations we extracted the known targets of 643 transcription factors from the TRANSFAC database [43] and associated the mRNA expressions of each transcription factor with its targets. The known targets in TRANSFAC are likely to be under-estimated, as only 10 transcription factors have valid expression data and contain more than 10 targets with valid expression data. Overall there are 155 transcription factor-known target pairs. Among them two transcription factors have significant associations with some of their known targets. MITF expression is associated with 4 of 9 targets: TYR, DCT, MLANA and TYRP1, and MYC expression is associated with 2 of 12 targets: CXCR4 and YBX1. The coverage rate is 3.87%.

#### Associations of CGH and karyotype data

To examine the effects of large-scale karyotype aberrations on DNA copy numbers we compared the segment CNVs inferred from the CGH data with the spectral karyotyping (SKY) data of NCI-60 cell lines [6]. Overall, copy numbers of the 49 chromosome segments derived from SKY and CGH data exhibit moderate correlations (Additional file 1, Figure S7). The means of intra-segment and all-vs-all correlation coefficients are 0.23 and 0 respectively (the Komolgorov-Smirnov p-value < 2 × 10^-19^). 14 of 49 segments have significant associations between the two datasets (log likelihood ratio ≥ 4.0, p-value ≤ 0.05). The two datasets are more strongly correlated when segments have extreme copy numbers. About one quarter of the data points have low (≤ 0.1) or high (≥ 0.9) normalized CGH values (793 and 718 of 2940 respectively). About half of the data points with ≤ 1 copy according to SKY also have low CGH values (100 of 224), whereas about one third of the data points with ≥ 3 copies according to SKY also have high CGH values (536 of 1623). The stronger correlation between SKY and CGH data on low copy number data points might result from the asymmetric influence of large-scale karyotype aberrations on copy numbers: deletions of a chromosome eradicate its DNA copies, while duplications do not necessarily increase the copy numbers if structural aberrations occur on the duplicated chromosomes.

The SKY data in NCI-60 provides lower resolution information about copy number variations than the CGH arrays, as only the cytogenetic bands of the abnormal karyotypes were reported. Therefore, we expected the segment CNVs derived from the SKY data would be less powerful to fit the gene expression data. Indeed, the segment CNVs derived from the SKY data invoked fewer associations than the CGH data: 1395 and 1526 gene expressions were associated with intra and internal segment CNVs derived from the SKY data, whereas 2165 and 1547 intra and inter segment associations with the CGH data were found.

### Expression data explained by unknown mechanisms

Many gene expressions are not explained by layer 1 or 2 models due to the incomplete information of the simple mechanisms listed above. Layer 3 models include the associations not constrained by known mechanistic links. In this study we considered the following types of layer 3 associations linking gene expressions with each type of molecular aberrations: segment CNVs on other chromosomes, mutations and DNA methylations of cancer-related genes obtained from OMIM. Overall, associations with external segment CNVs, mutations and DNA methylations explain a large number of expression data.

#### Associations with external segment CNVs

The expressions of 1547 genes are associated with the CNVs on other chromsomal segments. Table 1 reports the number of genes associated with each external segment CNV. Unlike internal segment CNVs, the number of genes associated with external segment CNVs is uncorrelated with the gene density and length of the segments (correlation coefficients 0.05 and 0.19 respectively). Some short segments accommodate a small number of genes but are associated with many genes on other loci. For instance, segment 11 (chromosome 4p 16.3) is only 1.17 Mb long but is associated with 119 external gene expressions.

It is difficult to directly validate the accuracy of associations with external segment CNVs as many possible mechanisms may underlie these associations. We considered one simple mechanism: copy number variations alter the expressions of a regulator gene on the segment, which modulates the expressions of its downstream genes on other segments. We identified these putative regulators based on the following criteria: (1)they are transcription factors, (2)they are related to cancer according to the OMIM database, (3)their expressions are explained by internal segment CNVs, (4)their expressions are associated with more than 20 genes on other chromosomes (see Additional file 1, Section *Identification of master regulators*, for the filtering procedure). 7 putative regulators were identified and reported in Table 3. Among them MITF, MYB and E2F3 contain information of downstream targets, and the association outcomes of MITF and MYB conform with the prior information.

**Table 3:** Putative transcription factor mediating inter-segment CNV associations. *N*: # genes on other chromosomes correlated with the putative regulator.

gene	desc	segment	*N*	gene	desc	segment	*N*
MITF	microphthalmia-associated TF	seg 9	246	MYB	v-myb myeloblastosis viral oncogene	seg 17	141
TCF7L2	TF7-like 2 (T-cell specific, HMG-box)	seg 24	101	E2F3	E2Ftranscription factor 3	seg 16	78
ZNF148	zinc finger protein 148	seg 10	69	GTF3A	general transcription factor IIIA	seg 33	69
MLF1	myeloid leukemia factor 1	seg 10	50				

**Figure 4:**
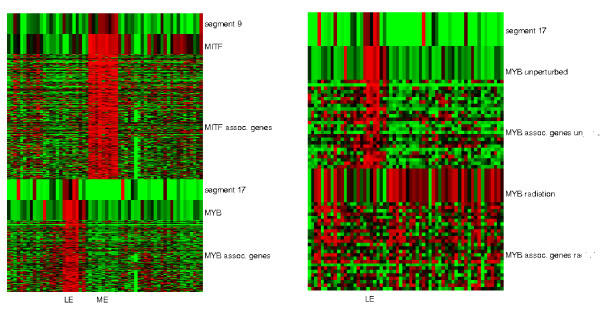
**Two examples of cis and trans acting effects of CNVs on gene expressions**. Left: Two modules are associated with putative regulators and external segment CNVs. The upper half of the heatmap shows the CNV of segment 9 (chr3p 13-14.2), the expression of MITF (chr3p 14.1) and associated genes on other chromosomes. The module is expressed in melanoma cell lines (ME). The lower half of the heatmap shows the CNV of segment 17 (chr6q 21-27), the expressions of MYB (chr6q 22) and associated genes on other chromosomes. The module is expressed in leukemia cell lines (LE). Right: mRNA expressions of the MYB module under unperturbed and radiation conditions. The upper half of the heatmap shows the CNV of segment 17, expressions of MYB and associated genes under the unperturbed condition. The lower half of the heatmap shows the expressions of MYB and associated genes under radiation.

MITF encodes a transcription factor that regulates the differentiation and development of melanocytes and pigment cell-specific transcription of the melanogenesis enzyme genes [50]. It is located on segment 9 (chromosome 3p 13-14.2). Both segment 9 CNV and MITF mRNA expression undergo melanoma-specific amplification. Moreover, MITF expression is associated with the 246 melanoma-specific gene expressions. This number far exceeds the 9 MITF targets from TRANSFAC. To gather a more complete information of MITF targets we extracted 106 experimentally validated MITF targets from [51] and found they intersected with our MITF-associated genes on 41 genes (hyper-geometric p-value < 5.1 × 10^-46^). Indeed, the causal chain from the amplification of chromosome 3p to the disregulation of MITF and its targets in NCI-60 was previously reported [31]. The upper left panel of Figure 4 shows the CNV of segment 9 and expressions of MITF and its associated genes.

MYB (c-myb) is a transcription factor involved in cell cycle progression, cell proliferation and differentiation in hematopoiesis [52]. It is located on segment 17 (chr6q 21-27). Amplifications of this oncogene cause its abnormal expressions in leukemia and other solid tumors ([52,53]). In NCI-60 both segment 17 CNV and MYB mRNA expression are elevated in leukemia cell lines. Thus MYB may mediate the associations of segment 17 with 141 leukemia-specific genes on other chromosomes. The lower left panel of Figure 4 shows the segment 17 CNV and expressions of MYB and its associated genes.

To further validate the associations with MYB we examined the mRNA expression data of NCI-60 cell lines exposed under radiation [54] and an expression data of 73 normal tissues [55]. 64 genes are associated with MYB expressions under the unperturbed condition and are probed in the radiation dataset. Among them 26 are also associated with MYB expressions in the radiation dataset (see Additional file 1, Section *Validation of master regulator-associated genes on the radiation data*, for the verification screening procedure). Conservation of MYB-associations between the two datasets is significant (hyper-geometric p-value ≤ 0.0024). Moreover, in an expression dataset of 73 normal tissues MYB and 20 of these 26 genes are highly expressed in blood and bone marrow samples. These lines of evidence suggest that the MYB-associated genes are either downstream targets or co-regulated partners of MYB. The right panel of Figure 4 shows the expression data of the MYB module under unperturbed and radiation conditions.

E2F3 is a member of the E2F transcription factor family that is over-expressed in bladder and prostate cancers [56]. We extracted 82 putative targets of E2F3 from knock-down experiments in bladder cancer [56] and found no overlap with the 78 E2F3 associated genes in NCI-60.

#### Associations with external gene mutations

We considered the associations of gene mutations and expressions in both directions: the mutation of one gene co-occurs with the up-regulation (positive) or down-regulation (negative) of another gene. Among the 24 mutated genes 6 are selected as they are mutated in more than 10 cell lines. Overall, 392 gene expressions are significantly associated with at least one of the 6 gene mutations in either direction. The left panel of Table 4 reports the number of gene expressions associated with each gene mutation. CDKN2A and TP53 mutations are associated with the highest numbers of gene expressions in both directions (78 positive and 70 negative associations for CDKN2A, 43 positive and 49 negative associations for TP53).

**Table 4:** Associations with gene mutations

mutated gene	*N*_1_	*N*_2_	gene	logratio	pvalue
CDKN2A	78	70	*MDM2	3.156	1.790e-02
TP53	43	49	*FDXR	3.065	< 1.000e-04
PIK3CA	49	12	*HSPB8	3.000	1.770e-02
PTEN	25	20	*THBS2	2.901	6.140e-02
KRAS	13	13	*CDKN1A	2.350	1.400e-03
APC	14	8	*TNFRSF10B	2.348	3.700e-03
			LTBR	2.296	< 1.000e-04
			EGFL7	2.163	1.500e-03
			*BAX	2.154	9.900e-03
			TMEM5	2.008	1.400e-03

Columns 1-3: number of gene expressions associated with each gene mutation in either direction. *N*_1_: # positively associated genes, *N*_2_: # negatively associated genes. Columns 4-6: top gene expressions negatively associated with TP53 mutation. Genes marked by asterisks are known TP53-regulated targets.

Only one mutated gene (TP53) is a transcription factor and has information about downstream targets. To validate the association results we extracted 45 known TP53-responsive genes confirmed by ChIP-Seq assays [57] and compared them to the genes positively and negatively associated with TP53 mutations. The 49 genes negatively associated with TP53 mutations have a significant enrichment with the 45 TP53 targets (intersect in 8 genes, hyper-geometric p-value < 3.32×10^-12^). Furthermore, 7 of the top 10 negative associations (sorted by log likelihood ratios) are known TP53 targets. For instance, the top candidate, MDM2, is a ubiquitin ligase and transcriptional target of TP53 [58]. The right panel of Table 4 reports these top-ranking negative associations with TP53 mutations. In contrast, genes positively associated with TP53 mutations are not enriched with known TP53 targets. Since the majority of TP53 mutations are loss-of-function (frameshift) mutations, our analysis indicates the effects of TP53 mutations on its activating targets are much more prominent.

#### Associations with external DNA methylation

We considered the negative associations of gene expressions with the DNA methylations of 125 signaling proteins or transcription factors. Some genes have similar methylation patterns, thus their associated expressions are highly overlapped. We obtained 14 clusters of methylated genes according to their associated expressions (see Additional file 1, Section *Clustering the DNA methylation data*). Additional file 1, Table S4 summarizes the information of these clusters.

**Figure 5:**
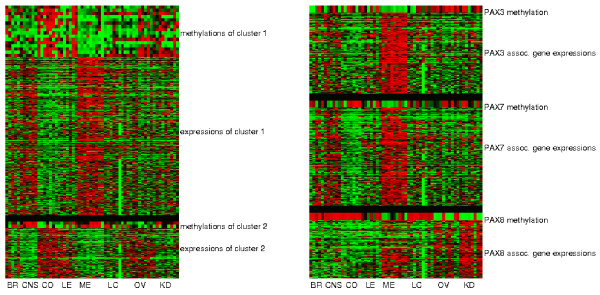
**Trans acting effects of DNA methylations on gene expressions**. Left: DNA methylation and gene expression data of the top 2 clusters of external methylation associations. From the top, the heatmap of the rows displays (1)methylation data of 16 genes in cluster 1, the height of each pixel is amplified ten times, (2)expression data of 490 genes associated with the methylated genes in cluster 1, (3)a black separator of the two clusters, (4)methylation data of 2 genes in cluster 2, the height of each pixel is amplified ten times, (5)expression data of 157 genes associated with the methylated genes in cluster 2. Right: DNA methylation and gene expression data pertaining to 3 PAX genes. From the top, the heatmap displays (1)PAX3 methylation data, (2)expression data of 115 genes associated with PAX3 methylation, (3)a black separator, (4)PAX7 methylation data, (5)expression data of 140 genes associated with PAX7 methylation, (6)a black separator, (7)PAX8 methylation data, (8)expression data of 83 genes associated with PAX8 methylation. Tissue abbreviations: BR:breast, CNS:central nervous system, CO:colon, LE:leukemia, ME:melanoma, LC:lung, OV:ovarian, KD:kidney.

Most of these clusters are associated with tissue-specific expression patterns. The left panel of Figure 5 shows the methylation and expression data of the top 2 clusters. Cluster 1 contains the highest number of methylated genes (16). These genes are hyper-methylated in colon cancer and leukemia cell lines and hypo-methylated in CNS and melanoma cell lines. They are negatively associated with 490 genes. Methylated genes in cluster 1 include proteins highly expressed in neural systems (CHGA, GABRB3, GAS7, FEV), strongly related to leukemia (TCL1A, FLT3, FLT4), involved in cell growth and proliferation (EGFR, WT1, SMO, IGF2, RET), and transcription factors involved in diverse or unknown functions (PAX3, PAX7, HOXC13, ZIM2). Cluster 2 contains 2 methylated genes BCR and BCL7A. They are hyper-methylated in CNS and melanoma samples and hypo-methylated in colon and leukemia samples. They are negatively associated with 157 genes.

7 methylated genes are associated with more than 40 gene expressions: ZIM2, PAX8, PAX7, PAX3, BCR, TCL1A, BCL7A. The right panel of Figure 5 shows the methylation patterns of 3 PAX genes and their associated expressions. Among them the expression profiles of PAX3-associated genes are consistent with its function. PAX3 is a regulator of MITF [59] and is involved in melanocyte-specific gene expressions ([60,61]). Thus in addition to amplification of segment 9 CNV the hypo-methylation of PAX3 in melanoma may also up-regulate the melanoma-specific gene expressions.

### Tissue-specific patterns of gene expressions and other aberrations

Due to their distinct origins from 9 tissue types, NCI-60 cell lines exhibit heterogeneous patterns of pathway/gene activities and molecular aberrations. In principle, associations between molecular aberrations and gene expressions may persist in multiple tissue types. For instance, TP53 mutations and the associations with its putative targets occur in most tissue types. However, to understand the mechanisms underlying each tumor type it is of interest to identify the tissue-specific patterns of molecular aberrations, gene expressions, and their associations.

We identified the tissue-specific patterns of 8094 gene expressions and sorted these patterns by the numbers of their constituent genes. For each pattern we then identified the dominant associations and enriched pathways and GO categories of the constituent genes. Detailed description of the identification of tissue-specific patterns is reported in Additional file 1, Section *Identifying tissue-specific patterns of gene expressions*. Table 5 lists the information of the top-ranking tissue-specific patterns. The first pattern contains 682 genes expressed in leukemia. Enrichment with generic processes of DNA replication, transcription, splicing and cell cycle indicates high activities of cell division and growth in leukemia cell lines. Many genes are associated with segment 17 (chr6q 21-27) CNV. From previous discussions some of these associations are likely to be mediated by MYB expressions. The second pattern contains 465 genes expressed in melanoma and are enriched with processes of melanosome, transporters and glycogen metabolism. Genes involved in melanocite differentiation are definitely expressed in melanoma. In addition, high activities of transport genes such as ABC transporters may account for strong drug resistance of melanoma cell lines (by analyzing the data from [23], results not shown). Dominant associations are the CNVs of segments 9 (chr3p 13-14.2) and 10 (chr3q 13.3-28) and PAX3 methylation. From previous discussions the associations with segment 9 CNV and PAX3 methylation are likely mediated by MITF expressions. Some enriched biological processes of the remaining patterns are consistent with the features of the corresponding tissues. Enrichment in cell cycle, DNA replication and translation genes reflects the high growth and division rates of leukemia and colon epithelial cells. Enrichment with transport and ion binding genes in kidneys and enrichment with estrogen receptors in ovarians are consistent with their tissue functions. However, some patterns do not possess prominent associations with observed aberrations, and other patterns lack putative regulators mediating the associations between segment CNVs and gene expressions.

**Table 5:** Top tissue-specific patterns of gene expressions and the associated molecular aberrations. CNV: copy number variation, mut: mutation, meth: DNA methylation.

tissues	# genes	aberrations	functions
leukemia	682	seg 17, 11 CNV	nuclear transport, splicing, cell cycle, RNA synthesis, DNA replication
melanoma	465	seg 9, 10 CNV, PAX3 meth.	glycogen metabolism, melanosome, transporter
colon	402	seg 33 CNV, APC TP16 mut.	liver development, cell cycle
CNS	334	seg 2, 49 CNV	-
kidney	251	-	apoptosis, transport, ion binding, cell shape
colon, leukemia	198	seg 11 and 33 CNV	DNA replication, translation
breast	147	seg 16 CNV	endoplasmic reticulum
ovarian	106	-	estrogen receptor

## Discussion

It is difficult to systematically evaluate the accuracy of the association outcomes since the mechanisms connecting molecular aberrations and gene expressions remain largely unknown. In this study we applied several procedures to validate the predictions. First, simulation studies indicate that high sensitivity and specificity of the links inferred from the layered models are robust against noise levels and gene numbers. Second, a conservative estimate yields FDRs of layer 1 and 2 models below 15% and the FDR of layer 3 models to be 38%. The ostensibly high FDR of layer 3 models is due to the large number of possible associations and strong dependency of the features in NCI-60 data. However, since the goal is an explorative analysis of possible links explaining gene expressions instead of identifying the features segregating a specific phenotype (e.g., survival duration), we can apply stringent cutoffs and external knowledge to trim the spurious associations. Third, the coverage rates (# association links/# known mechanistic links) in layer 1 and 2 models vary considerably by types of association links. The heterogeneous coverage rates reflect the diverse characteristics of the mechanisms. For instance, frame-shift mutations almost certainly disrupt mRNA synthesis hence yield strong associations with gene expressions. In contrast, the association of a transcription factor-target pair may be weak since their regulatory interaction is present in only certain cell types. Fourth, top-ranking layer 3 associations with single genes (mutations, methylations) possess more supporting evidence from previous studies than associations with chromosomal segments. This is because the majority of previous studies report associations between genes (e.g., co-expression) rather than associations between genes and chromosomal segments. Fifth, the inferred layer 3 models involved in a few transcription factors with rich information are compared with their known targets. Among the 3 transcription factors (MITF, MYB, E2F3) which may mediate the associations from their segment CNVs to the expressions of other chromosomes, associations from two of them (MITF, MYB) have experimental supports according to previous studies. Layer 3 associations with mutations of the only transcription factor (TP53) are also enriched with its known targets.

Many questions arise from our analysis results and demand further investigations. It is unclear why some gene expressions are modulated by the copy numbers of their local segments but others are not. Other factors determining transcript levels (e.g., chromatin structure, protein and DNA modification, trans-regulation by other transcription factors, RNA degradation) may mask or dilute the effect of copy numbers. In-depth analysis on the alterations of gene regulation are required to investigate the effects from other factors.

Associations in this study are derived from observational data alone. Causal information is partially encoded in the mechanistic assumptions of the model (e.g., mutation of a gene affects its expression, but mutation is not altered by gene expression). Yet perturbation data such as knock-outs/downs and knock-ins are required in order to fully discern the causal chains connecting certain aberrations. For instance, to validate that E2F3 mediates the associations from segment 16 CNV to gene expressions on other chromosomes, we can knock-down E2F3 and observe the responses of the putative targets. In addition to validation, the layered models can also include perturbation data at the inference stage.

We chose NCI-60 data as a testing stone for the layered modeling framework since it is one of the most comprehensive cancer data in terms of the types of probed features. Despite its rich information NCI-60 data suffers from several limitations. First, it covers cell lines from diverse origins. Heterogeneous patterns of gene/pathway disregulation may add difficulty in analyzing the data. Second, NCI-60 data lacks normal tissues as negative controls. The "abnormal" changes in gene expressions, copy number variations and DNA methylations are all relative to other cancer cell lines in the panel. Therefore, fluctuations of molecular aberrations and phenotypic responses are more likely to reflect tissue-specific variations than cancer-normal cell differences. Third, utility of cancer cell lines in studying the biology of primary/metastasized tumors and drug discovery is still under debate. These intrinsic drawbacks of NCI-60 cell lines limit their applications in cancer biology. Nevertheless, the layered modeling framework can be applied to other comprehensive datasets of primary tumors with negative controls (e.g., the TCGA data).

One potential problem of reconstructing the statistical/causal relations of molecular aberrations in tumor datasets is the genotypic/phenotypic heterogeneity of cells. Unlike cell lines, most tumors have heterogeneous patterns of gene mutations, copy number variations, epigenetic modifications and gene expressions over a population of cells. It remains a challenge to deconvolve the data generated from the mixed population.

## Conclusion

Diverse molecular aberrations and phenotypic responses constitute high-dimensional signatures of cancer cells. It is also important to reconstruct the statistical and causal relations of the molecular aberrations and phenotypic responses. In this study we proposed a layered modeling framework to identify the associations of gene mutations, copy number variations, DNA methylations, mRNA and protein expressions on NCI-60 cancer cell lines. We sequentially applied three layers of models with increasing complexity and uncertainty to fit the gene expression data. Layer 1 models use local aberrations to explain the expressions on the same loci. Layer 2 models use nonlocal aberrations with known mechanistic links to explain the expressions on different loci. Layer 3 models use nonlocal aberrations with missing mechanistic links to fit the expressions on different loci.

Sensitivity analysis from simulated data, false discovery rates, coverage rates relative to known mechanistic links, literature search, and comparisons with the known targets of several well-studied transcription factors all verify the association outcomes from the layered models. Specifically, our analysis identifies the following prominent associations on NCI-60 data. First, about 70% of the protein expressions are significantly associated with their mRNA expressions, substantiating the consistency of mRNA and protein expression data. Second, several gene expressions are associated with composite local aberrations. For instance, the protein expressions of CDKN2A are repressed by either frame-shift mutations or DNA methylations. Third, amplification of chromosome 6q in leukemia is likely to elevate the expression of MYB, and the downstream targets of MYB on other chromosomes are up-regulated accordingly. Fourth, amplification of chromosome 3p and hypo-methylation of PAX3 together may elevate MITF expression in melanoma, which may up-regulate the downstream targets of MITF. Fifth, mutations of TP53 are negatively associated with its direct target genes. The results justify the utility of the layered models for the incoming flow of large-scale, integrated cancer genomic data.

## List of abbreviations

CNV: Copy Number Variation; CGH: Comparative Genomic Hybridization; SKY: Spectral Karyotyping; FDR: False Discovery Rate; BNT: Bayesian Network Structural Learning; CNS: Central Nervous System.

## Authors' contributions

CHY is the single author and is responsible for all the work in the paper.

## Supplementary Material

Additional file 1 An integrated analysis of molecular aberrations in NCI-60 cell lines – Supplementary Information, Figures and Tables. Click here for file
