# Supplementary material for: An integrated analysis of molecular aberrations in NCI-60 cell lines
Source: BMC Bioinformatics. 2010 Oct 6;11:495. doi: 10.1186/1471-2105-11-495 (PMC2984587; doi:10.1186/1471-2105-11-495)
Supplement: Additional file 1 — An integrated analysis of molecular aberrations in NCI-60 cell lines – Supplementary Information, Figures and Tables. [file 1471-2105-11-495-S1.PDF]

An integrated analysis of molecular aberrations in NCI-60 cell lines – Supplementary Information, Figures and Tables

Methods of data processing, analysis, and simulation experiments

Data processing

Our modeling framework is based on logistic regression models of discrete random variables. Like other discrete models (such as Boolean and Bayesian networks), this class of simple models can represent complex yet intuitive relations of genes such as their combinatorial interactions. Since most NCI-60 data in this study constitute continuous values, quantization of data into discrete values is needed. Quantization with a "hard threshold" suffers from two drawbacks: the problem of determining the threshold value and the loss of information in continuous data. To overcome these limitations we applied probabilistic quantization to convert measurement values into the probabilities of discrete states. Information in the continuous data is preserved in the probabilities of states, and the discrete quantization is softened by probability assignments.

Features with numerical values – mRNA and protein expressions, copy number variations, and DNA methylation – were treated as discrete random variables with three possible states – up-regulation, down-regulation and no change. Features with categorical values – mutations – were treated as binary random variables. For each dataset, denote z_{ij} the observed value of gene i on cell line j, and x_{ij} its discrete hidden state. The following procedures convert each z_{ij} into a probability vector $(P(x_{ij} = -1), P(x_{ij} = 0), P(x_{ij} = 1))$.

1. Discard the measurements of a gene if there are ≥ 5 missing data points among the 60 cell lines.
2. Rank-transform z_{ij} into the cumulative distribution function (CDF) value $y_{ij} \in [0, 1]$. For the datasets reporting relative values (cDNA, CGH and protein expression data), rank transformation is applied to the entire matrix. For the datasets reporting absolute values (Affymetrix data), each feature is rank-transformed separately. This is because we want to capture the relative variation of a feature across different cell lines instead of comparing the values of distinct features. DNA methylation data are scaled in $[0, 1]$ thus need not to be rank-transformed.
3. Convert y_{ij} into a probability vector $(P(x_{ij} = -1), P(x_{ij} = 0), P(x_{ij} = 1))$ with a specific quantization function. Intuitively, a data point with a low CDF value is more likely to be down-regulated ($P(x_{ij} = -1)$ is high), and a data point with a high CDF value is more likely to be up-regulated ($P(x_{ij} = 1)$ is high). This intuition is translated into the requirements that a quantization function is monotonic and maps $y_{ij} = 0$ into $P(x_{ij} = -1) = 1$ and $y_{ij} = 1$ into $P(x_{ij} = 1) = 1$. We chose polynomial functions f_γ and $\bar{f}_\gamma$ as the quantization curves.

$$\begin{aligned} P(x_{ij} = 1 | y_{ij}, \gamma) &= f_\gamma(y_{ij}) \equiv y_{ij}^\gamma. \\ P(x_{ij} = -1 | y_{ij}, \gamma) &= \bar{f}_\gamma(y_{ij}) \equiv (1 - y_{ij})^\gamma. \\ P(x_{ij} = 0 | y_{ij}, \gamma) &= 1 - P(x_{ij} = 1 | y_{ij}, \gamma) - P(x_{ij} = -1 | y_{ij}, \gamma). \end{aligned} \quad (1)$$

Parameter γ controls the "soft thresholds" of assigning x_{ij} to be +1 or -1. A higher γ lifts the threshold on y_{ij} (and $1 - y_{ij}$) of calling the hidden state x_{ij} to be 1 (and -1). Thus a higher γ raises $P(x_{ij} = 0)$ and reduces $P(x_{ij} = \pm 1)$. Supplementary Figure 1 shows examples of several quantization curves.

4. Integrate the transformed values over a family of polynomial quantization functions. Quantization results are sensitive to γ values. To reduce the bias induced by a specific quantization function we assigned weights (prior) on f_γ functions and averaged the outputs over the quantization functions. In this work we chose an exponential prior $e^{-(\gamma-1)}$ and restrict $\gamma \in [1, \infty)$. The averaged quantization outputs are:

$$\begin{aligned} P(x_{ij} = 1|y_{ij}) &= \int_1^\infty e^{-(\gamma-1)} f_\gamma(y_{ij}) d\gamma &&= \tfrac{y_{ij}}{1-\log y_{ij}}. \\ P(x_{ij} = -1|y_{ij}) &= \int_1^\infty e^{-(\gamma-1)} \bar{f}_\gamma(y_{ij}) d\gamma &&= \tfrac{1-y_{ij}}{1-\log(1-y_{ij})}. \\ P(x_{ij} = 0|y_{ij}) &= 1 - P(x_{ij} = 1|y_{ij}) - P(x_{ij} = -1|y_{ij}). \end{aligned} \tag{2}$$

The exponential prior $e^{-(\gamma-1)}$ was chosen for the following reasons. First, large γ values are penalized because they assign the probability mass to $x_{ij} = 0$ for most y_{ij} values. An exponential prior naturally penalizes large γ values. Second, it ensures the existence of the integrals in equation 2. Third, the requirements that $P(x_{ij} = 1|y_{ij} = 1) = 1$ and $P(x_{ij} = -1|y_{ij} = 0) = 1$ are satisfied. Fourth, the most justified single value of γ is $\hat{\gamma} = \frac{\log 3}{\log 2}$ because it assigns an equal probability ($\frac{1}{3}$) for each state when the input CDF $y_{ij} = 0.5$. The marginal quantization curves are indeed similar to the quantization curves generated by $\hat{\gamma}$ (Supplementary Figure 1).

Mutation data are converted into binary values: $P(x_{ij} = 1) = 1$ if gene i is mutated in cell line j, and $P(x_{ij} = 0) = 1$ otherwise. In some part of the analysis we set $P(x_{ij} = 1) = 1$ only if a frame-shift mutation occurs on gene i and cell line j.

The SKY/M-FISH data of karyotypes specify the chromosome locations of karyotypic aberrations – amplifications, deletions and translocations – of each chromosome in each cell line. To compare the CGH and SKY/M-FISH data we constructed the normalized copy number profiles of each chromosomal segment over the 60 cell lines from each type of data. Each chromosome was partitioned into multiple segments according to the CGH data (see subsection Inferring segment CNVs from CGH data from the main text and subsection Partitioning a chromosome into segments according to CGH data from the supplement). The copy number profiles from the CGH data were the CDF values inferred from the data of all probes on the segments (equations 3 and 4 in the main text). The copy number profiles from the SKY/M-FISH data were obtained by counting the karyotypic aberrations on each chromosomal segment and cell line. A chromosomal segment has two copies in a normal karyotype. Amplification or translocation on a chromosomal segment adds its copy number by one, and deletion on a chromosomal segment subtracts its copy number by one.

To speed up model selection we filtered the candidate associations between aberration features and gene expressions by their correlation coefficients. Associations with the correlation coefficients below the threshold were not considered for model selection. The following threshold values were applied: internal segment CNVs and external mutations: 0.3, external segment CNVs: 0.35, external methylations: 0.4.

Combination of cDNA and Affymetrix data

Consistency of cDNA and Affymetrix data was verified by comparing the correlation coefficients of the expression profiles of the two datasets. The cDNA and Affymetrix data intersect in 5251 genes. We computed the correlation coefficients between cDNA and Affymetrix data on each of the 5251 genes. As a comparison we also calculated the correlation coefficients between all valid genes of the two datasets. The distribution of the intra-gene correlation coefficients is significantly higher than that of the background distribution (Supplementary Figure 2): the mean of the intra-gene correlation coefficients is 0.4 (the mean of the background distribution is 0), and the Komolgorov-Smirnov p-value $< 10^{-100}$.

To utilize both cDNA and Affymetrix data we adopted the following procedures to combine the two datasets.

1. 2857 genes have consistent cDNA and Affymetrix expression data (correlation coefficient ≥ 0.4). We chose 0.4 as the threshold for the following reasons. First, the intra-gene correlation coefficients of cDNA and Affymetrix data have a bimodal distribution (Supplementary Figure 2, the blue solid curve). 0.4 can be viewed as the boundary of separating the "correlated" and "uncorrelated" pairs. Second, the intra-gene correlation coefficients of mRNA and protein data also centers around 0.4. Third, correlation coefficients often under-estimate nonlinear dependency. Among the 2857 genes 2451 of them have log likelihood scores ≥ 3.0 and permutation p-values ≤ 0.01. We selected these 2857 genes and used the cDNA data to represent their mRNA expressions, since by replacing the cDNA with Affymetrix data 2221 of 2226 associations were retained.
2. 1374 genes have valid cDNA data but no valid Affymetrix data. Without information to reject them we incorporated these cDNA data in the mRNA expression dataset.
3. 7817 genes have valid Affymetrix data but no valid cDNA data. 3954 of them have strongly correlated Affymetrix expression profiles (mean correlation coefficient 0.71). Because this large cluster of genes have almost identical expression profiles and are not strongly associated with any molecular aberrations, it is efficient to discard them instead of incurring model selection for each gene separately.

Overall, the combined dataset contains 4231 cDNA and 3863 Affymetrix gene expressions.

Partitioning a chromosome into segments according to CGH data

Spatial dependency of CNVs is manifested from the measurements of 219 genes on a Comparative Genomic Hybridization (CGH) array. Supplementary Figure 3 shows the CNV correlation coefficients of the CGH probes sorted by chromosome coordinates. Clearly, most strongly correlated pairs (bright red pixels) appear near the diagonal entries, indicating that spatially adjacent genes tend to have correlated CNVs across the 60 cell lines. Comparison of the distributions of correlation coefficients of adjacent genes versus the entire gene set also supports the spatial dependency (Komolgorov-Smirnov p-value $< 8.44 \times 10^{-296}$).

To extrapolate the CGH data of sparse probes into the surrounding regions we devised a recursive algorithm to partition each chromosome into correlated segments. In brief, the CGH data of all probes on the same segment are treated as instantiations of a common hidden variable (a naive Bayes model, Friedman, Geiger and Goldszmidt 1997).

The algorithm iteratively partitions a segment that optimizes the joint likelihood and stops when further partitions do not improve the likelihood score.

1. Quantize the CDF values of the CGH data into three levels $(0, \pm 1)$ using thresholds 0.4 and 0.6.
2. Denote $S = \{\pi_1, \cdots, \pi_m\}$ a collection of consecutive probes and $X_S = \{x_1, \cdots, x_m\}$ their quantized CGH data. The log likelihood function $L(X_S)$ of a naive Bayes model of X_S is computed by the following procedures:
   (a) The values of the hidden variable y_S are the consensus of $x_1, \cdots, x_m$ on each sample.
   (b) Count the prior frequencies of the hidden variable $p_a = \frac{N(y_S=a)}{n}$, where n is the sample size.
   (c) Count the transition frequencies $q_{b|a} \equiv \frac{1}{m} \sum_{i=1}^{m} \frac{N(x_i=b, y_S=a)}{N(y_S=a)}$.
   (d) The log likelihood of the naive Bayes model is $L(X_S) = \sum_{a=-1}^{1} [N(y_S = a) \log(p_a) + \sum_{b=-1}^{1} \sum_{i=1}^{m} N(y_S = a, x_i = b) \log(q_{b|a})]$.
3. Initially set S to be the probes on the entire chromosome.
4. Iteratively incur the function $partition(S)$:
   (a) Evaluate $L(X_S)$.
   (b) Find the binary partition (S_1, S_2) of S that maximizes $L(X_{S_1}) + L(X_{S_2})$.
   (c) Stop and return S if $L(X_S) \geq L(X_{S_1}) + L(X_{S_2})$.
   (d) Otherwise incur $partition(S_1)$ and $partition(S_2)$.

Simulation studies

We compared the performance of the layered models with other methods on 10 simulated datasets. In each simulation a model of 10, 50 or 100 genes was created by randomly assigning each gene expression to be affected by one or two of the following aberrations: local mutation and DNA methylation, CNV of its chromosome, and the expression of a master transcription factor. Boolean functions relating the input and output variables were established accordingly. Trinary discrete values $(0, \pm 1)$ of the input variables were randomly generated, and those of the expression variables were determined by the input values and the Boolean functions. The trinary output values were than converted to $(0, \pm 2)$. A zero-mean, independent Gaussian noise with standard deviations 0.2, 0.3 or 1.0 was added to the discrete expression values. There are 9 combinations of gene numbers and noise levels in the experimental setting.

Three different methods were applied to the artificial data: the layered logistic regression models, Bayesian network structure learning (the Baye Net Toolbox (BNT) Matlab codes), and the Matlab k-means clustering function (k=4). The layered logistic regression model was applied to identify the aberration variables explaining each expression data. The p-value cutoff 0.05 was used in model selection. The model graph was generated by connecting the assigned aberration variables with each expression variable. We also applied the module network learning program (Segal et al. 2003) to the datasets of 10 genes. However, since the codes for automatically running the simulated experiments are not available (a stand-alone program Genomica implements the clustering and learning of module networks) and sensitivities on the 10-gene datasets are substantially lower than other three methods, we did not include the results of the module networks in the paper.

Simulated data were quantized with thresholds -1 and +1 before feeding into the Bayesian network structure learning codes. A matlab program K2 in BNT learns the structure of a Bayesian network with a fixed topological order. The Bayesian network structure learned from each dataset was determined by model averaging over 10 random permutations of topological orders. An edge e was present in the consensus graph G if e was present in more than 40% of the learned structures.

The kmeans function in Matlab was also applied to the simulated data. The number of clusters was fixed to 4 and Euclidean distance was chosen as the distance metric. The model graph was generated by connecting the aberration and expression variables in the same cluster.

Model graphs generated by each method were compared with the artificial model underlying the simulated data. True positives were the edges present in the true model and selected by the learning algorithm, and true negatives were the edges absent in the true model and not selected by the learning algorithm. Sensitivity ($\frac{\text{\# true positives}}{\text{\# positives in the true model}}$) and specificity ($\frac{\text{\# true negatives}}{\text{\# negatives in the true model}}$) of the predictions over 20 datasets were calculated.

Estimation of false discovery rates

False discovery rates measure the expected ratio of the numbers of false positives and positive calls (Benjamini and Hochberg 1995):

$$FDR = E\{\frac{\text{\# false positives}}{\text{\# positive calls}}\}. \quad (3)$$

Benjamini and Hochberg proposed a Bonferroni-type correction on positive calls to control the false discovery rate. This procedure is probably too conservative for the top-ranking features. In this work we replace equation 3 with two related quantities using permutation tests. The first quantity evaluates the expected false positive number according permutation tests over the number of positive calls from the empirical data (Storey and Tibshirani 2006).

$$FDR1 = \frac{E\{\text{\# false positives}\}}{\text{\# positive calls}}. \quad (4)$$

The second quantity evaluates the 99 percentile of false positive number according to permutation tests over the number of empirical positive calls (Korn et al. 2003).

$$FDR2 = \frac{\arg\max_k \{P(\text{\# false positives} \leq k) \geq 0.99\}}{\text{\# positive calls}}. \quad (5)$$

FDR2 is more conservative since the 99 percentile of false positive number is always greater than or equal to its mean. Both quantities demand the probability distribution of positive calls under the null hypothesis. We obtained this distribution by implementing the following permutation tests over 1000 random trials. In each trial, we randomly permuted the data points of each feature separately. We then incurred the layered model selection on the permuted data and counted the number of significant associations that pass the thresholds on log likelihood scores and p-values. The distribution of false positive calls was approximated by the histogram of the number of significant associations.

We then plugged this distribution in equations 4 and 5 to evaluate the two types of FDRs. In addition, we counted the associations of each layer separately and reported the FDRs of all the associations and of those from each layer.

Functional enrichment analysis

We grouped genes according to the types of molecular aberrations that explain their expression data. There are 34 gene groups associated with internal segment CNVs, 43 gene groups associated with external segment CNVs, 15 gene groups associated with mutations and 20 gene groups associated with DNA methylations. For each group we calculated the hyper-geometric p-values of enrichment in 4822 Gene Ontology (GO) categories and 889 pathways from Reactome, BioCarta, and NCI-Nature. 24 gene groups associated with internal segment CNVs, 19 gene groups associated with external segment CNVs, 8 gene groups associated with mutations and 2 gene groups associated with DNA methylations were enriched with at least one functional category. Supplementary Table 2 shows the enriched functional categories for each gene group.

Identification of master regulators

Master regulators are the transcription factors/signaling proteins which mediate the associations of segment CNVs with gene expressions on other chromosomes. To qualify as a master regulator the following criteria must hold for gene g.

1. g is a transcription factor or signaling protein according to its GO annotation. In other words, its GO terms contain the keywords "transcription factor", "signaling" or "signal transduction".
2. g is related to cancer according to the OMIM database. In other words, its OMIM annotation contains keywords "cancer", "tumor", "oncogene", "leukemia" or "carcinoma".
3. g is associated with the CNV of its internal segment s according to layered 2 models.
4. The expression of g is correlated with at least 20 genes on other chromosomes (correlation coefficient ≥ 0.3).

We also performed functional enrichment analysis on the genes associated with each master regulator. The results are shown in Supplementary Table 2.

Validation of master regulator-associated genes on the radiation data

To reduce spurious associations with the master regulators we checked whether co-expression of the putative targets of each master regulator was retained in the expression data of the NCI-60 cell lines under the γ-ray radiation (Amundson et al. 2008). The correlation coefficient distribution q of the entire dataset (5343 genes) was treated as the background distribution. For each master regulator, we identified the putative targets by associating their gene expressions in the mRNA data under the unperturbed condition (see *Identification of master regulators*), and evaluated the correlation coefficient distribution p of the putative targets in the radiation data. Co-expression is retained in the radiation data if p is significantly greater than q (Komolgorov-Smirnov p-value < 0.05).

Identification of the putative targets of TP53 mutations

Despite TP53 mutations are not tissue specific, some genes associated with TP53 mutations in layer 3 models have strong tissue-specific patterns. To eliminate the effect of tissue types we tested whether the associations with TP53 mutations can be explained away by tissue types. The "activity" of gene g in a tissue type was inferred from the g expressions of the corresponding cell lines. The inference returns an identical value in all the data points of the same tissue type and follows equation 2 in the Supplement and equation 4 in the main text. Denote TP53 mutational states as μ, tissue-specific activity of gene g as t_g, and g expression as e_g. We built two logistic regression models explaining e_g: M_1 uses t_g alone to fit e_g: $P_1(e_g|\mu, t_g) \equiv P_1(e_g|t_g)$, and M_2 uses both μ and t_g to fit e_g: $P_2(e_g|\mu, t_g) \equiv P_2(e_g|\mu, t_g)$. Putative targets in the right column of Table 4 in the main text are sorted by their log-likelihood ratios R_{21} (equation 5 in the main text).

Clustering the DNA methylation data

We clustered the DNA methylation data according to the overlap of their associated mRNA expressions. For each gene in the DNA methylation data we identified its associated mRNA expressions according to the layer 3 models. A graph G of the methylated genes was constructed. Two methylated genes were adjacent if their associated mRNA expressions were overlapped in ≥ 10 genes. Methylated genes in the same connected component of G were clustered together.

Identifying tissue-specific patterns of gene expressions

We implemented a matrix-decomposition method to identify the tissue-specific patterns of gene expressions. Denote $y(t)$ the expression profile of a gene where t is the index of cell lines. We constructed 9 tissue-specific expression profiles $x_i(t)$:

$$x_i(t) = \begin{cases} 1 \text{ if cell line } t \text{ belongs to tissue } i, \\ 0 \text{ otherwise.} \end{cases} \tag{6}$$

The $x_i(t)$'s represent perfect tissue-specific profiles. Each $y(t)$ can be written as a linear combination of $x_i(t)$'s plus an error term:

$$y(t) = \sum_i \lambda_i x_i(t) + e(t). \tag{7}$$

λ_i's are obtained by minimizing the squared error of the approximation:

$$\mathcal{L}(\Lambda) = \sum_t (y(t) - \sum_i \lambda_i x_i(t))^2. \tag{8}$$

By taking partial derivatives of $\mathcal{L}$ with respect to each λ_i the optimizer is the solution of a linear matrix equation:

$$\begin{pmatrix} \sum_t x_1(t)x_1(t) \cdots \sum_t x_1(t)x_9(t) \\ \vdots\vdots\vdots\vdots \\ \sum_t x_9(t)x_1(t) \cdots \sum_t x_9(t)x_9(t) \end{pmatrix} \begin{pmatrix} \lambda_1 \\ \vdots \\ \lambda_9 \end{pmatrix} = \begin{pmatrix} \sum_t x_1(t)y(t) \\ \vdots \\ \sum_t x_9(t)y(t) \end{pmatrix}. \tag{9}$$

Coefficients λ_i's specify the relative importance of each tissue in fitting a gene expression profile. We are interested in the profiles with high coefficients in a small number of tissues (≤ 3) . For each decomposition $y(t) = \sum_i \lambda_i x_i(t)$ the following filtering criteria were implemented to identify these profiles:

1. Sort λ_i's in a decreasing order.
2. Find the minimum i such that $\lambda_i \geq 1.5\lambda_{i+1}$.
3. If $i \leq 3$ then report the top i tissues. Otherwise do not report a tissue.

These criteria assign each expression profile a discrete pattern of tissue-specific expressions. For instance, CD53 is highly expressed in leukemia and silent in all other tissues. Expression profiles which do not pass the filtering criteria are either silent in all tissues or expressed in heterogeneous tissue types.

We then counted the number of expression profiles assigned to each tissue-specific pattern and sorted these patterns accordingly. Functional enrichment analysis was performed on the genes belonging to the top-ranking patterns, and the dominant molecular aberrations associated with those genes were reported.

Additional analysis results

Global accuracy validation of inferred associations

For layer 1 and 2 models, we evaluated the coverage rates of significant associations relative to the possible mechanistic links of each type of aberration. Coverage rates reveal false negatives from the data. For instance, 68 genes contain valid mRNA and protein expression data, and 46 of them manifest significant associations between mRNA and protein expressions. Thus the coverage rate is 68%.

It is difficult to estimate false positives and false negatives of layer 3 associations due to the large number of possible associations. Therefore, we solicited the top-ranking associations according to likelihood scores and performed literature search. We categorized the results of literature search into 3 classes: (1)Previous studies provide direct evidence for the associations. For instance, knock-out experiments or ChIP-seq assays identify the targets of a transcription factor, (2)Previous studies contain indirect evidence for possible associations. For instance, two associated genes are co-expressed in specific tissues of non-NCI-60 datasets, (3)There is no evidence of associations from pubmed keyword search.

Supplementary Table 1 reports the literature search results for top-ranking layer 3 associations. 10 of top 50 associations with external segment CNVs possess indirect evidence. The genes are either on the same chromosomes of the segments or are putatively regulated by the genes on the segment. For instance, the associations of PTPRC and CD53 mRNA expressions with segment 17 CNV are the top 2 entries. Several previous studies suggest that PTPRC and CD53 are associated with MYB, which is located on segment 17. The third entry is an association of CD200 mRNA expression with segment 9 CNV. CD200 and segment 9 are located on different arms of the same chromosome (chr3q and chr3p respectively). 5 of top 50 associations with external gene mutations possess direct evidence, and 16 associations possess indirect evidence. All the associations with direct evidence are pertaining to TP53 mutations due to the rich literature on TP53 targets. Associations supported by indirect evidence often constitute genes co-expressed

or co-mutated in the same samples. For instance, the association of CAV1 mRNA expression and CDKN2A mutation is the second entry of the top 50 associations. Combined loss of CAV1 and CDKN2A can synergistically enhance cell proliferation and tumorigenesis. 13 of top 50 associations with external DNA methylations possess indirect evidence.

mRNA and protein expression data are consistent

To validate the consistency of mRNA and protein expression data we calculated the intra-gene correlation coefficients and all-vs-all correlation coefficients between the two datasets. Supplementary Figure 5 shows the distributions of intra-gene correlation coefficients and the all-vs-all background. The Komolgorov-Smirnov p-value $< 1.26 \times 10^{-27}$. The mean of intra-gene correlations is 0.4 and the mean of the background distribution is 0.

The CGH probes of the same inferred segments exhibit coherent CNV values

To justify the partitioning algorithm we demonstrated that the CGH probes located on the same inferred segments were strongly correlated compared to the background distribution. Supplementary Figure 4 shows the correlation coefficient distributions of the CGH probes on the same segments and on distinct segments. The two distributions display strong disparity. The means of the intra-segmental and inter-segmental correlation coefficients are 0.65 and 0 respectively, and the Komolgorov-Smirnov p-value $< 10^{-260}$.

CGH and FISH data are moderately consistent

We compared the CNV profiles of each segment inferred from the CGH and FISH data and found they were moderately correlated. Supplementary Figure 7 shows the intra-segment and all-vs-all correlation coefficient distributions between CGH and FISH datasets. The means of intra-segment and all-vs-all correlation coefficients are 0.23 and 0 respectively, and the Komolgorov-Smirnov p-value $< 2 \times 10^{-19}$.

Some tissue-specific expression profiles are associated with the CNVs of their segments

In NCI-60 we observe 11 chromosome segments have tissue-specific CNV changes. These CNV changes are associated with many tissue-specific expression changes, suggesting that some tissue-specific expression patterns may result from the copy number changes of the chromosome segments. For instance, segment 2 (chromosome 1p 13-33) is amplified in the central nervous system (CNS) cell lines, and 59 genes on the segment also exhibit CNS-specific expression patterns. Similarly, segment 9 (chromosome 3p 13-14.2) has high copy numbers in melanoma and is associated with 11 melanoma-specific expression patterns on the segment. Segment 17 (chromosome 6q 21-27) has high copy numbers in leukemia and is associated with 30 leukemia-specific gene expressions on the segment. Supplementary Figure 6 shows the tissue-specific CNVs and expressions explained by internal segment CNVs.

Tumor phenotypes arising from the copy number changes of oncogenes or tumor suppressors are reported (Frohling and Dohner 2008). We extracted 4291 cancer-related genes from the OMIM database (OMIM) and identified 452 cancer-related genes associated with the CNVs on their chromosome segments. Abnormal expressions of these genes

caused by copy number changes are likely to induce cancer-related phenotypes. For example, NRAS and RAP1A are highly expressed in CNS and are associated with the CNV of segment 2 (chromosome 1p 13-33). Both genes are members of the Ras signaling pathway, thus their up-regulation in CNS may elevate the activity of the pathway. Supplementary Table 3 reports the cancer-related genes associated with segment CNVs.

Tissue-specific expressions are associated with external DNA methylation We considered the negative associations of gene expressions with the DNA methylations of 125 signaling proteins or transcription factors. Some genes have similar methylation patterns, thus their associated expressions are highly overlapped. We obtained 14 clusters of methylated genes according to their associated expressions. Supplementary Table 4 summarizes the information of these clusters.

Legends of supplementary figures

Fig. 1. Quantization curves converting a CDF value into a probability vector $(P(x=-1), P(x=0), P(x=1))$. Abscissa is the CDF value and ordinate shows the probabilities $P(x=\pm 1, 0)$. Blue, red and green curves are the quantization functions for $P(x=1)$, $P(x=-1)$, $P(x=0)$ respectively. Solid lines: marginal curves obtained by integrating over a family of polynomial curves. Dash-dotted lines: polynomials with $\gamma = 1$. Dotted lines: polynomials with $\gamma = 10$. Dashed lines: polynomials with $\gamma = \frac{\log 3}{\log 2}$, which assigns an equal probability of $P(x=-1), P(x=0), P(x=1)$ when the CDF is 0.5.

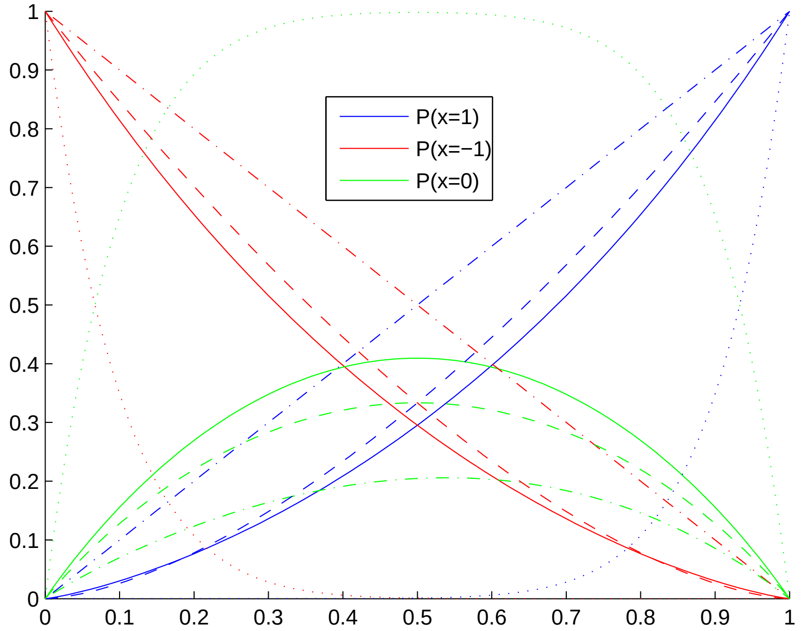

Fig. 2. Correlation coefficient distributions of mRNA expressions between cDNA and Affymetrix data. The solid blue line shows the correlation coefficients of the same genes between the two datasets. The dashed red line shows the correlation coefficients between all probes of the two datasets.

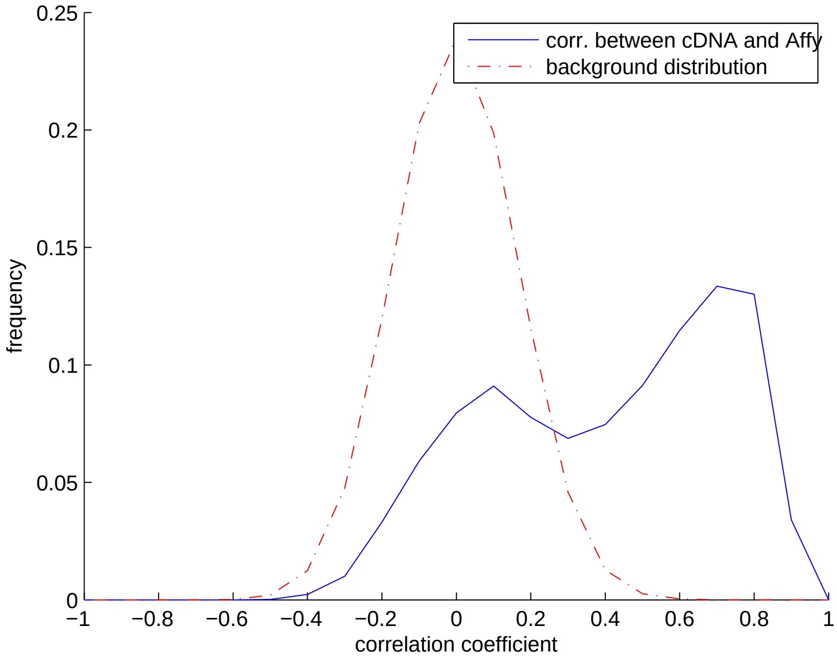

Fig. 3. Spatial dependency of CNV probes on the CGH array. 219 probes are sorted by chromosome coordinates and correlation coefficients of their CGH measurements across 60 cell lines are plotted. Red pixels denote positive correlations and green ones denote negative correlations. Each cyan box marks the probes on the same chromosome.

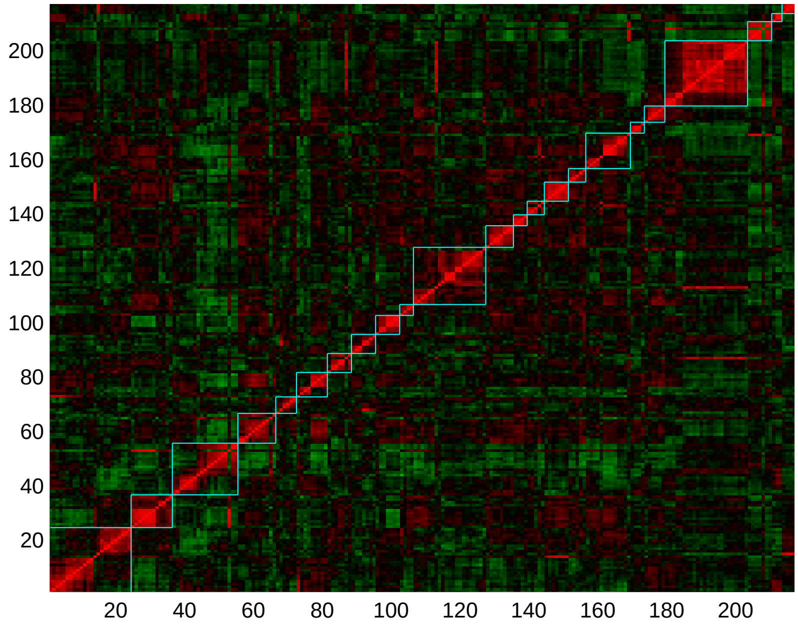

Fig. 4. Correlation coefficient distributions of the CNV values of the CGH probes on inferred segments. The solid blue line shows the correlation coefficients of the CGH probes on the same segments. The dashed red line shows the correlation coefficients of the CGH probes on the distinct segments.

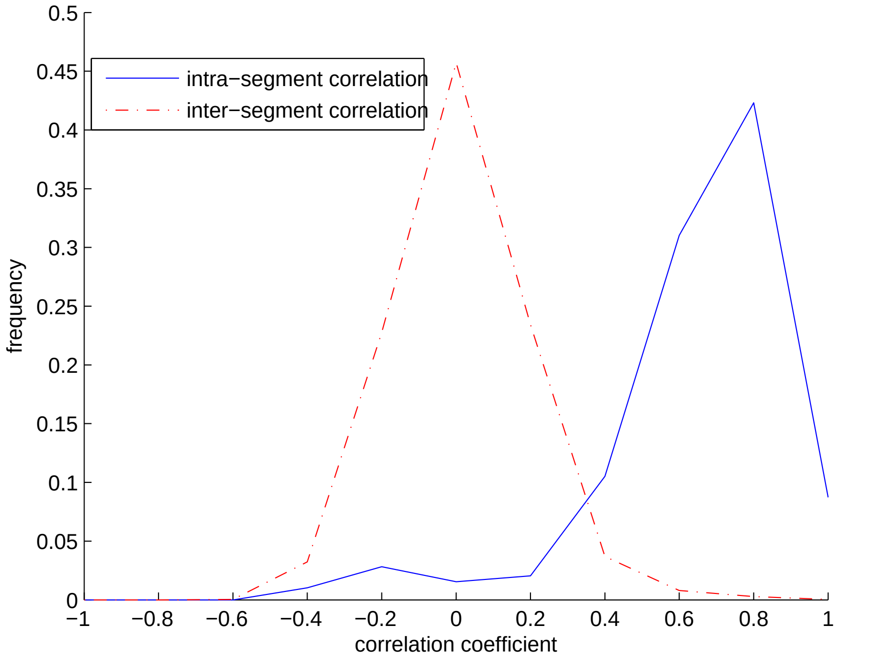

Fig. 5. Correlation coefficient distributions of mRNA and protein expressions. The solid blue line shows the correlation coefficients of the two datasets in the same genes. The dashed red line shows the background distribution between all probes of the two datasets.

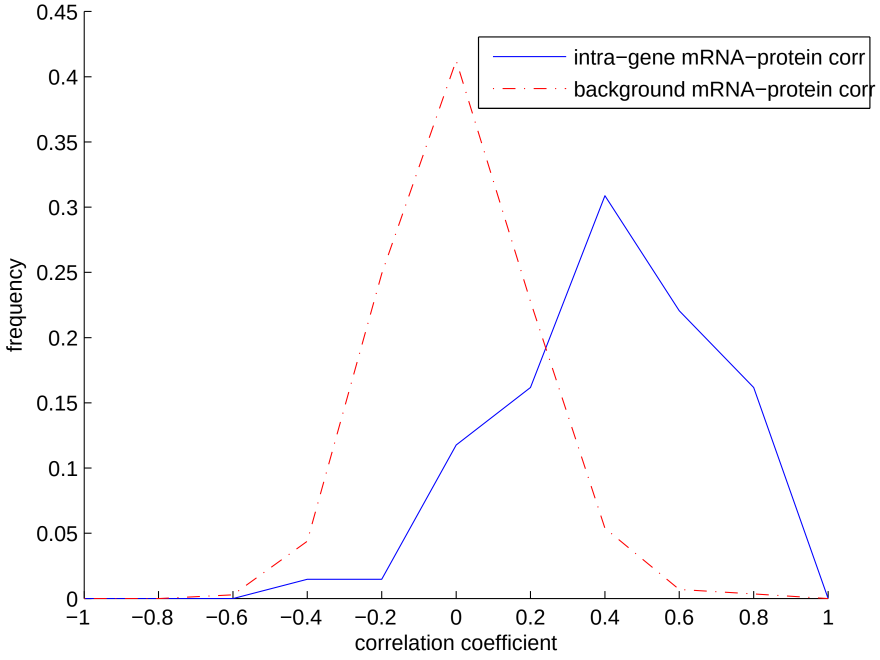

Fig. 6. Tissue-specific segment CNVs and their associated mRNA expressions. The elongated profile on the top of each segregated band shows the segment CNV. The remaining profiles in the band show the mRNA expressions of the associated genes. The data are normalized into the cumulative distribution values in $[0, 1]$. High CDF values are coded by red and low CDF values are coded by green.

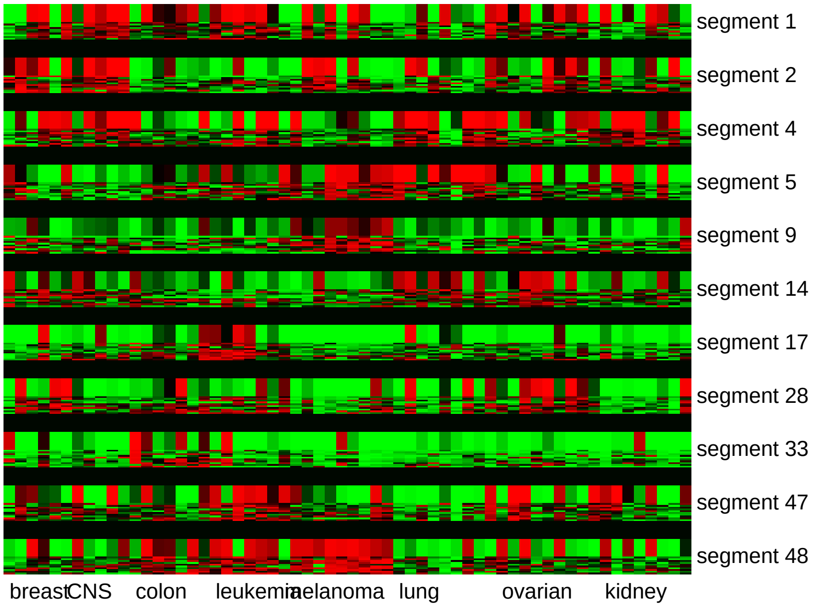

Fig. 7. Correlation coefficient distributions of the CNVs inferred from CGH and SKY data. The solid blue line shows the correlation coefficients of CGH and SKY data on the same segments. The dashed red line shows the correlation coefficients of CGH and SKY data between all segments.

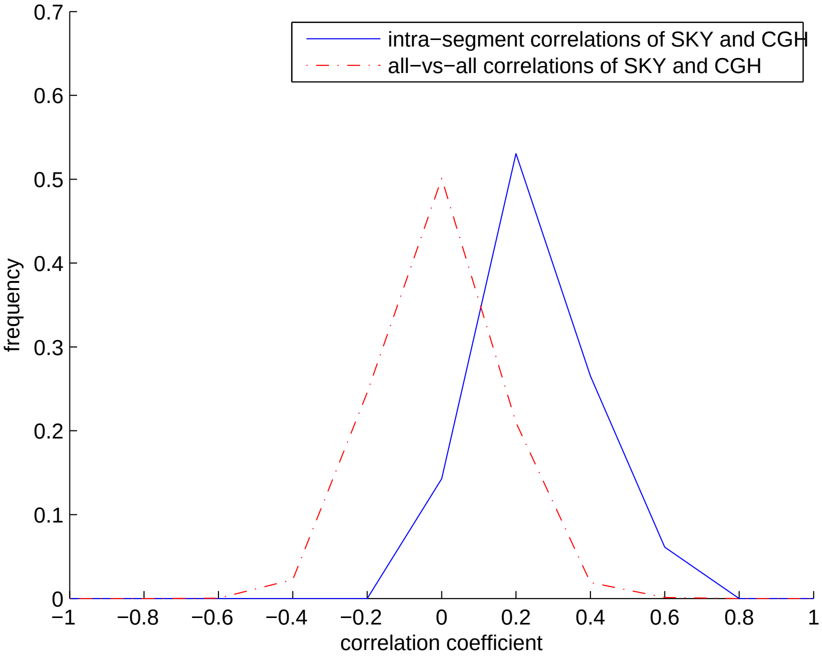

Legends of supplementary tables

Table 1. Literature search results of top-ranking layer 3 associations.

Table 2. Functional enrichments of gene sets explained by each type of molecular aberration.

Table 3. Cancer genes explained by intra-segment CNVs.

Table 4. Summary of clusters of methylated genes.

Literature search results of top-ranking layer 3 associations.

external segment CNV

Gene	Aberration	Evidence	PMIDs
PTPRC	segment 17 CNV	indirect	16757688, 15296653, 12691915, 9949159, 9808556, 9215748
CD53	segment 17	indirect	18464117
CD200	segment 9 CNV	indirect	12853143
S100P	segment 33 CNV	unknown	
LRRC1	segment 33 CNV	unknown	
C2orf32	segment 9 CNV	unknown	
TACSTD1	segment 33 CNV	unknown	
TIMP3	segment 9 CNV	indirect	9693046
ACOT2	segment 45 CNV	unknown	
CDH1	segment 33 CNV	unknown	
MAP7	segment 33 CNV	unknown	
CKMT1B	segment 33 CNV	unknown	
LILRB2	segment 34 CNV	unknown	
NDUFA9	segment 33 CNV	unknown	
FABP7	segment 9 CNV	unknown	
PAICS	segment 17 CNV	unknown	
ZNF497	segment 34 CNV	unknown	
IMPA2	segment 33 CNV	unknown	
C19orf25	segment 41 CNV	unknown	
STIL	segment 2 CNV	indirect	12681356, 12199779, 11390401
SMARCA5	segment 11 CNV	indirect	18505340
GPM6B	segment 9 CNV	unknown	
CMTM4	segment 24 CNV	unknown	
LSM6	segment 11 CNV	indirect	18042180
RELA	segment 16 CNV	unknown	
WT1	segment 17 CNV	indirect	9361007, 7559553, 10465027, 11001926
AKAP1	segment 33 CNV	unknown	
CLSTN1	segment 43 CNV	unknown	
HCLS1	segment 17 CNV	unknown	
TUBB2A	segment 2 CNV	unknown	
LOC646417	segment 8 CNV	unknown	
IL18	segment 24 CNV	indirect	16325763, 15358834.
LEF1	segment 48 CNV	unknown	
EXOSC9	segment 11 CNV	unknown	
IVD	segment 34 CNV	unknown	
COPG	segment 9 CNV	unknown	
ANAPC10	segment 11 CNV	indirect	19657357, 17031559, 15611179
ELP4	segment 11 CNV	unknown	
ATP1A1	segment 9 CNV	unknown	
ZNF430	segment 1 CNV	unknown	
CBLN2	segment 7 CNV	unknown	
APOL1	segment 36 CNV	unknown	

FOXJ3	segment 1 CNV	unknown
SCC-112	segment 11 CNV	unknown
SMC1B	segment 29 CNV	unknown
SDHAL2	segment 26 CNV	unknown
AAA1	segment 33 CNV	unknown
RPL9	segment 37 CNV	unknown
LOC388344	segment 37 CNV	-
RPL14	segment 8 CNV	-

external mutation

Gene	Aberration	Evidence	PMIDs
CLEC2B	PTEN+		unknown
CAV1	CDKN2A+	indirect	15044451, 12460915
ITGA3	CDKN2A+	indirect	17638301
CRABP2	PIK3CA+	unknown	
FXYD3	CDKN2A-	unknown	
ERBB3	CDKN2A-	indirect	18225590, 18061531, 17409975
JUP	PIK3CA+	unknown	
MRPL37	PIK3CA+	unknown	
DSP	CDKN2A-	unknown	
RTN4	CDKN2A+	unknown	
TACSTD2	PIK3CA+	unknown	
GDA	PIK3CA+	unknown	
SPINT2	PIK3CA+	unknown	
FLJ34306 CDKN2A-	unknown		
TACSTD1	CDKN2A-	unknown	
RAB8B	CDKN2A+	indirect	20096196
ZNF266	TP53+	unknown	
MDM2	TP53-	direct	19649205
STX1A	CDKN2A+	indirect	9950615
LGMN	CDKN2A-	unknown	
FDXR	TP53-	direct	16413492, 18572087, 14559799
S100A4	KRAS+	unknown	
MSN	CDKN2A+	indirect	19555429, 12460915
CAV2	CDKN2A+	indirect	20007452
HSPB8	TP53-	direct	18418217
TFPI	CDKN2A+	indirect	15629771
TPP1	PTEN-	unknown	
LIMK1	CDKN2A+	indirect	19018287, 11795508, 11301474, 9787135
TOP2A	KRAS+	unknown	
CLEC2B	CDKN2A+	unknown	
PLSCR1	KRAS-	unknown	
CDH1	CDKN2A-	indirect	19450526
LYZ	APC+	unknown	
ERGIC3	CDKN2A-	unknown	
YKT6	KRAS-	unknown	

TRDMT1	CDKN2A+	indirect	19680556, 11230735
MAL2	PIK3CA+	indirect	17001163, 16317585, 15761962
LYZ	CDKN2A-	unknown	
ZNF415	TP53+	indirect	17055453
FOXO3A	TP53+	indirect	18824006, 15067352
MAP1B	CDKN2A+	unknown	
PAICS	PTEN+	unknown	
SLFN12	CDKN2A+	unknown	
CDKN1A	TP53-	direct	16413492, 9822382
TNFRSF10B	TP53-	direct	16413492, 17881637
PPL	PIK3CA+	unknown	
DBNL	STK11+	unknown	
ZNF583	TP53+	unknown	
DNER	CDKN2A+	indirect	19405089
F2R	CDKN2A+	indirect	19664933, 10362519

external methylation

Gene	Aberration	Evidence	PMIDs
GPX2	TCF1-	unknown	
ALDH1A1	TCF1-	indirect	9882450, 9168903
TNC	TCL1A-	unknown	
CTSB	TCL1A-	unknown	
NDUFA9	TCF1-	unknown	
IGFBP5	ZIM2-	unknown	
PLCB4	PAX8-	indirect	10505771
SLC29A2	BCR-	unknown	
TRIB2	ZIM2-	unknown	
JAZF1	TCL1A-	unknown	
KIAA1026	PAX8-	unknown	
HSPB2	PAX8-	indirect	15693623
UGT2B7	PAX8-	indirect	19945756
SLC26A2	PAX8-	unknown	
LAMP2	TCL1A-	unknown	
ATP1B3	PAX7-	unknown	
HBM	PAX3-	unknown	
NCOA5	PAX7-	indirect	19953635
LSR	BCR-	unknown	
ITGB3	PAX8-	indirect	19699783, 15161828, 11937757, 11520860, 11179695, 10954416, 9740657, 9334279, 8983174, 7573028
ST7	PAX3-	unknown	
COPB2	ZIM2-	unknown	
SPP1	PAX8-	unknown	
PPP2R3A	PAX8-	unknown	
PTN	TCL1A-	unknown	
TIMP3	TCL1A-	unknown	

MAGEA3	PAX3-	indirect	15817820
TXNDC5	PAX7-	indirect	14965345
MAGEA12	ZIM2-	unknown	
RNF14	HOXC13-	unknown	
ATP5L	BCR-	unknown	
SLC24A1	PAX8-	unknown	
ATP10A	PAX8-	unknown	
LRRC61	BCL7A-	unknown	
S100P	TCF1-	indirect	11395380
SH3BP5	ZIM2-	unknown	
DBNDD2	BCR-	unknown	
FABP7	ZIM2-	unknown	
PIK3R3	GABRB3-	indirect	16890252, 16086687, 12598604
SOX12	FLT3-	indirect	19656071
CLGN	CD38-	unknown	
ACSL5	BCL7A-	unknown	
TMEM168	PAX3-	unknown	
PTPN3	BCL7A-	indirect	15897551
KIAA0090 IGF2-	unknown		
SGK	PAX3-	unknown	
SLC1A4	PAX3-	unknown	
ARG99	ZIM2-	unknown	
PPIL2	LCK-	indirect	12088411, 11438176, 10612659, 8283032
BDH1	LCK-	unknown	

Enriched functional classes of the gene groups explained by each type of molecular aberration.

segment CNV

segment_ind	location	nintra	GOs/pathways	ninter	GOs/pathways
1	chr1p 31.00-36.20	143	protein binding| Wnt Signaling-Noncanonical Pathway|	58	transcription factor activity|Assembly of the ORC complex at the origin of replication| CDC6 association with the ORC:origin complex| E2F-enabled inhibition of pre-replication complex formation|
2	chr1p 13.00-33.00	59	neural tube closure|	149	-
3	chr1q 21.00-23.00	0	-	0	-
4	chr1q 23.00-43.00	76	-	48	-
5	chr2p 12.00-24.10	82	-	80	-
6	chr2q 23.00-24.00	23	-	21	-
7	chr3p 24.20-25.00	18	ruffle|	34	-
8	chr3p 14.30-21.30	50	RNA binding|	100	Lysine catabolism|
9	chr3p 13.00-14.20	11	-	179	folic acid transporter activity|Toll Like Receptor 9 (TLR9) Cascade|
10	chr3q 13.30-28.00	81	inactivation of gsk3 by akt causes accumulation of b-catenin in alveolar macrophages| skeletal muscle hypertrophy is regulated via akt-mtor pathway|	162	proton transport|hydrogen ion transporting ATPase activity, rotational mechanism|y branching of actin filaments|
11	chr4p 16.30-16.30	29	-	172	protein binding|RNA splicing| pseudouridylate synthase activity| APC/C:Cdc20 mediated degradation of mitotic proteins| APC-Cdc20 mediated degradation of Nek2A| Inactivation of APC/C via direct inhibition of the APC/C complex| Viral Messenger RNA Synthesis| Cdc20:Phospho-APC/C mediated degradation of Cyclin A| mRNA Splicing - Major Pathway|
12	chr4q 21.00-31.21	0	-	0	-
13	chr4q 31.00-35.10	0	-	0	-

14	chr5q 35.10-35.10	20	ER-Golgi intermediate compartment| unfolded protein binding|	38	-
15	chr5q 31.00-31.10	38	ubiquitin-dependent protein catabolic process|	21	-
16	chr6p 21.20-21.30	87	nucleus| protein binding| identical protein binding| protein serine/threonine kinase activity|	89	death receptor binding|
17	chr6q 21.00-27.00	30	MAP kinase kinase kinase activity|	82	histone deacetylase binding|
18	chr7q 21.00-22.00	16	-	47	-
19	chr7q 21.00-31.00	40	role of pi3k subunit p85 in regulation of actin organization and cell migration| how does salmonella hijack a cell| y branching of actin filaments| M/G1 Transition| DNA Replication Pre-Initiation| Removal of licensing factors from origins| Assembly of the pre-replicative complex| Cell Cycle, Mitotic| Orc1 removal from chromatin| G1/S Transition|	52	phosphoprotein phosphatase activity| melanocyte development and pigmentation pathway|
20	chr8p 11.00-22.00	36	transcription regulator activity|	25	-
21	chr8q 11.00-13.00	48	inositol-1(or 4)-monophosphatase activity|	6	-
22	chr9q 22.30-22.30	13	-	45	-
23	chr9q 34.00-34.10	34	protein phosphatase type 2A regulator activity| GTP hydrolysis and joining of the 60S ribosomal subunit| L13a-mediated translational silencing of Ceruloplasmin expression| Peptide chain elongation| Eukaryotic Translation Termination| Viral mRNA Translation| Formation of a pool of free 40S subunits| Cap-dependent Translation Initiation|	82	transcription elongation factor complex|
24	chr10q 11.20-26.00	86	-	138	positive regulation of mitosis|positive regulation of epidermal growth factor receptor activity|prion pathway|
25	chr11p 15.40-15.50	17	-	52	-
26	chr11p 13.00-15.50	34	-	27	angiogenesis|
27	chr11q 11.00-13.00	0	-	0	-

28	chr11q 21.00-23.20	44	-	43	actin filament binding|
29	chr11q 13.00-14.00	11	-	80	-
30	chr11q 13.00-13.00	2	-	40	chromosome segregation|
31	chr12q 13.00-13.30	41	-	77	-
32	chr12q 14.30-24.30	68	regulation of progression through cell cycle| ubiquitin-dependent protein catabolic process| cell to cell adhesion signaling|	52	-
33	chr13q 12.30-21.20	52	-	92	mitochondrion|endothelial cell migration|
34	chr14q 11.20-32.32	150	DNA repair| polyadenylation of mrna|	69	-
35	chr15q 22.00-26.10	94	-	62	isoprenoid biosynthetic process|
36	chr16p 13.10-13.30	40	Displacement of DNA glycosylase by APE1|	51	-
37	chr16q 22.10-24.30	41	protein catabolic process| Packaging Of Telomere Ends|	61	-
38	chr17p 13.00-13.30	47	JNK cascade| nucleolus| cell cortex|	80	-

39	chr17q 11.20-22.00	85	chromatin binding| androgen receptor signaling pathway| histone deacetylase binding| thyroid hormone receptor binding| vitamin D receptor binding| proteasome complex (sensu Eukaryota)| proteasome complex| SCF-beta-TrCP mediated degradation of Emi1| Removal of licensing factors from origins| CDK-mediated phosphorylation and removal of Cdc6| Autodegradation of Cdh1 by Cdh1:APC/C| Vif-mediated degradation of APOBEC3G| Vpu mediated degradation of CD4| Cdc20:Phospho-APC/C mediated degradation of Cyclin A| SCF(Skp2)-mediated degradation of p27/p21| Degradation of beta-catenin by the destruction complex| APC/C:Cdh1 mediated degradation of Cdc20 and other APC/C:Cdh1 targeted proteins in late mitosis/early G1| Orc1 removal from chromatin| CDT1 association with the CDC6:ORC:origin complex| Ubiquitin-dependent degradation of Cyclin D1| Switching of origins to a post-replicative state| Ubiquitin Mediated Degradation of Phosphorylated Cdc25A| APC/C:Cdc20 mediated degradation of Securin| Host Interactions of HIV factors|	51	DNA replication factor C complex| Polymerase switching on the C-strand of the telomere| Repair synthesis of patch ~27-30 bases long by DNA polymerase| Repair synthesis for gap-filling by DNA polymerase in TC-NER| Polymerase switching|
40	chr17q 22.00-23.20	32	positive regulation of gene-specific transcription| egf signaling pathway| il 4 signaling pathway| growth hormone signaling pathway| cbl mediated ligand-induced downregulation of egf receptors pathway| trka receptor signaling pathway|	45	-
41	chr18p 11.20-11.31	17	membrane fusion|	74	Chk1/Chk2(Cds1) mediated inactivation of Cyclin B:Cdk1 complex|

42	chr19q 12.00-13.40	82	RNA binding| positive regulation of cell motility| RNA processing| basic mechanisms of sumoylation|	36	-
43	chr20p 13.00-13.00	23	-	24	-
44	chr20p 11.23-13.00	11	-	46	-
45	chr20q 13.00-13.31	8	-	22	enzyme activator activity|
46	chr20q 11.20-13.20	9	-	16	-
47	chr21q 22.10-22.30	41	protein import into nucleus|	31	-
48	chr22q 12.20-13.10	48	mitochondrion| mRNA Splicing - Minor Pathway| mRNA Splicing - Major Pathway|	145	-
49	chr23q 11.20-28.00	76	-	30	cytokinesis|

external gene mutation

mutated_gene_ind	mutated_gene	#genes	dir		GOs/pathways
1	TP53	45	positive		integral to peroxisomal membrane|mRNA catabolic process, nonsense-mediated decay|nucleocytoplasmic transporter activity|
1	TP53	55	negative		protein homooligomerization|caspase activation|positive regulation of cell migration|hypoxia and p53 in the cardiovascular system|p53 signaling pathway|
8	CDKN2A	101	positive		caveolar membrane|wound healing| Signaling events mediated by VEGFR1 and VEGFR2| Signal attenuation|
8	CDKN2A	86	negative		hexokinase activity|
38	PTEN	29	positive		-
38	PTEN	21	negative		-
39	APC	16	positive		-
39	APC	9	negative		-
40	BRAF	11	positive		-
42	PIK3CA	51	positive		peptide cross-linking|sulfotransferase activity|

42	PIK3CA	17	negative	-
227	KRAS	15	positive	multicellular organismal development| chromatin binding|
227	KRAS	14	negative	vesicle targeting|
3122	STK11	10	positive	peroxisome|DNA repair|
3122	STK11	9	negative	-

external DNA methylation

methylated_gene_ind	methylated_gene	naffected	GOs/pathways
14087	ZIM2	63	-
7138	PAX8	61	-
10500	PAX7	52	protein-tyrosine sulfotransferase activity| peptidyl-tyrosine sulfation| inhibition of matrix metalloproteinases|
6346	PAX3	48	-
58	BCR	47	-
123	TCL1A	46	-
1422	BCL7A	43	protein binding| intracellular signaling cascade|
1190	PPARG	30	-
1420	CHGA	30	-
6166	TCF1	29	-
13514	HOXC13	29	-
7139	FLT4	23	-
672	CD38	22	-
2212	SYK	20	-
16	EGFR	17	-
2215	GABRB3	15	-
12687	MAFB	13	-
112	LCK	11	-
13122	ZNF331	11	-
3133	RET	10	-

master regulator<br>master gene	# regulated	GOs/pathways
MITF	246	copper ion binding(5.945e-04)|melanosome(1.943e-08)|melanin biosynthetic process from tyrosine(2.492e-04)|phosphoprotein phosphatase activity(7.736e-04)|
PARD6A	168	regulation of transcription from RNA polymerase II promoter(9.231e-04)|translation factor activity, nucleic acid binding(6.305e-04)|translation(2.401e-09)|RNA binding(3.179e-04)|condensed chromosome(3.058e-04)|integral to nuclear inner membrane(6.076e-04)|cytosolic large ribosomal subunit (sensu Eukaryota)(4.691e-04)|structural constituent of ribosome(5.408e-09)|GTP hydrolysis and joining of the 60S ribosomal subunit(4.149e-05)|L13a-mediated translational silencing of Ceruloplasmin expression(3.866e-05)|Peptide chain elongation(2.122e-05)|Eukaryotic Translation Termination(2.122e-05)|Viral mRNA Translation(1.807e-05)|Formation of a pool of free 40S subunits(1.807e-05)|Cap-dependent Translation Initiation(3.866e-05)|

BMPR1A	151	positive regulation of mitosis(8.903e-04)|positive regulation of epidermal growth factor receptor activity(2.705e-04)|positive regulation of phosphorylation(5.375e-04)|epidermal growth factor receptor activity(2.705e-04)|positive regulation of epithelial cell proliferation(4.623e-05)|positive regulation of MAP kinase activity(2.705e-04)|prion pathway(5.270e-04)|agrin in postsynaptic differentiation(3.716e-04)|

MYB	141	nucleus(6.222e-04)|protein binding(4.685e-05)|regulation of transcription from RNA polymerase II promoter(4.745e-04)|RNA binding(5.184e-07)|chromatin remodeling(9.959e-04)|RNA processing(1.696e-04)|androgen receptor signaling pathway(1.127e-04)|transcription initiation from RNA polymerase II promoter(8.348e-05)|mediator complex(3.083e-04)|mRNA processing(7.114e-05)|double-stranded DNA binding(5.050e-05)|condensed chromosome(2.444e-04)|DNA unwinding during replication(8.051e-04)|ligand-dependent nuclear receptor transcription coactivator activity(1.521e-04)|heterogeneous nuclear ribonucleoprotein complex(1.091e-05)|RNA polymerase II transcription mediator activity(1.148e-04)|poly(A) binding(8.051e-04)|thyroid hormone receptor binding(3.769e-04)|vitamin D receptor binding(3.975e-05)|negative regulation of cytokine and chemokine mediated signaling pathway(8.197e-05)|control of gene expression by vitamin d receptor(8.348e-05)|Transport of Mature mRNA derived from an Intron-Containing Transcript(5.050e-05)|Viral Messenger RNA Synthesis(1.126e-05)|Processing of Capped Intron-Containing Pre-mRNA(4.779e-06)|mRNA Splicing - Major Pathway(1.169e-08)|Elongation of Intron-Containing Transcripts and co-transcriptional mRNA splicing(1.985e-05)|mRNA 3'-end processing(6.830e-06)|Cleavage of Growing Transcript in the Termination Region(2.269e-05)|

PRDX3	122	androgen binding(8.883e-04)|positive regulation of mitosis(5.953e-04)|positive regulation of epidermal growth factor receptor activity(1.804e-04)|prion pathway(2.912e-04)|
NOTCH2	119	-
ACVR1B	118	-
TCF7L2	101	positive regulation of mitosis(4.081e-04)| positive regulation of epidermal growth factor receptor activity(1.235e-04)|multi-drug resistance factors(5.157e-06)|
NCOA4	96	positive regulation of epithelial cell proliferation(9.491e-04)|
CTBP1	95	RNA polymerase II transcription factor activity(3.040e-04)|mRNA processing(1.099e-05)| paraspeckles(3.159e-05)|
TIAL1	94	-
CBLB	90	-
GSK3B	78	catalytic activity(4.097e-04)|
E2F3	78	-
DVL3	78	inhibition of matrix metalloproteinases(7.089e-04)|
GTPBP1	72	-
ZNF148	69	-
GTF3A	69	mitochondrion(9.836e-04)|
BCL10	69	-
CRK	68	-
PLCE1	68	regulation of cell adhesion(3.784e-04)|
MAPK1	65	melanosome(3.551e-04)|role of erk5 in neuronal survival pathway(1.681e-04)| phospholipids as signalling intermediaries(1.028e-05)|
BRCA2	64	potassium:chloride symporter activity(8.943e-05)|

PIK3CB	63	hydrogen ion transporting ATPase activity, rotational mechanism(9.898e-05)|
ZIC1	61	-
ERCC5	56	Viral Messenger RNA Synthesis(8.771e-04)|
RYK	51	-
MLF1	50	-
MNAT1	49	-
TYMS	48	G2/M transition of mitotic cell cycle(3.187e-04)|mitotic spindle organization and biogenesis(3.187e-04)|APC/C:Cdc20 mediated degradation of Cyclin B(9.187e-04)|Phosphorylation of the APC/C(9.187e-04)|
HMGB1	48	-
LMO4	47	collagen type V(2.808e-05)|
RAP1A	47	endoplasmic reticulum(1.648e-04)|
NFKB2	44	-
LTB4R	44	-
MAPKAPK3	42	-

List the cancer genes explained by each intra-segment CNV.

segment	segment location	gene name	gene function	correlation	logratio	pvalue
1	chr1p 31.00-36.20	EIF2C3	eukaryotic translation initiation factor 2C, 3	0.61	12.34	1.60E-003
1	chr1p 31.00-36.20	GNL2	guanine nucleotide binding protein-like 2 (nucleolar)	0.57	11.63	3.00E-004
1	chr1p 31.00-36.20	AOF2	amine oxidase (flavin containing) domain 2	0.53	10.92	2.97E-006
1	chr1p 31.00-36.20	YTHDF2	YTH domain family, member 2	0.52	10.71	5.20E-003
1	chr1p 31.00-36.20	TTC4	tetratricopeptide repeat domain 4	0.44	9.58	3.00E-003
1	chr1p 31.00-36.20	RAD54L	RAD54-like (S. cerevisiae)	0.42	9.27	1.67E-002
1	chr1p 31.00-36.20	S100PBP	S100P binding protein	0.42	9.26	7.40E-003
1	chr1p 31.00-36.20	CDC20	cell division cycle 20 homolog (S. cerevisiae)	0.4	9.16	1.70E-003
1	chr1p 31.00-36.20	FAF1	Fas (TNFRSF6) associated factor 1	0.4	9.11	2.07E-002
1	chr1p 31.00-36.20	DVL1	dishevelled, dsh homolog 1 (Drosophila)	0.39	8.97	1.90E-003
1	chr1p 31.00-36.20	HDAC1	histone deacetylase 1	0.38	8.91	1.17E-002
1	chr1p 31.00-36.20	PTP4A2	protein tyrosine phosphatase type IVA, member 2	0.28	8.76	3.00E-004
1	chr1p 31.00-36.20	RRAGC	Ras-related GTP binding C	0.36	8.75	2.82E-002
1	chr1p 31.00-36.20	DFFA	DNA fragmentation factor, 45kDa, alpha polypeptide	0.36	8.72	3.98E-002
1	chr1p 31.00-36.20	RCC1	regulator of chromosome condensation 1	0.34	8.67	7.84E-002
1	chr1p 31.00-36.20	SDHB	succinate dehydrogenase complex, subunit B, iron sulfur (Ip)	0.41	8.63	9.00E-003
1	chr1p 31.00-36.20	TCEB3	transcription elongation factor B (SIII), polypeptide 3 (110kDa, elongin A)	0.42	8.5	8.90E-003
1	chr1p 31.00-36.20	EIF2C1	eukaryotic translation initiation factor 2C, 1	0.36	8.43	6.00E-004
1	chr1p 31.00-36.20	LEPRE1	leucine proline-enriched proteoglycan (leprecan) 1	0.32	8.29	5.06E-002
1	chr1p 31.00-36.20	SNIP1	Smad nuclear interacting protein 1	0.32	8.28	7.82E-002
1	chr1p 31.00-36.20	MYCBP	c-myc binding protein	0.31	8.19	4.27E-002
1	chr1p 31.00-36.20	CTPS	CTP synthase	0.32	8.12	8.10E-003
1	chr1p 31.00-36.20	KIF1B	kinesin family member 1B	0.36	8.11	1.27E-002
1	chr1p 31.00-36.20	FUSIP1	FUS interacting protein (serine/arginine-rich) 1	0.39	8	7.40E-003

1	chr1p 31.00-36.20	ZMPSTE24	zinc metallopeptidase (STE24 homolog, S. cerevisiae)	0.25	7.67	5.68E-002
1	chr1p 31.00-36.20	FRAP1	FK506 binding protein 12-rapamycin associated protein 1	0.32	7.55	3.33E-002
1	chr1p 31.00-36.20	EPS15	epidermal growth factor receptor pathway substrate 15	0.27	7.43	2.68E-002
1	chr1p 31.00-36.20	CDKN2C	cyclin-dependent kinase inhibitor 2C (p18, inhibits CDK4)	0.19	6.31	1.19E-001
1	chr1p 31.00-36.20	PTCHD2	patched domain containing 2	0.03	5.69	1.01E-001
2	chr1p 13.00-33.00	NRAS	neuroblastoma RAS viral (v-ras) oncogene homolog	0.56	12.74	1.00E-004
2	chr1p 13.00-33.00	RAP1A	RAP1A, member of RAS oncogene family	0.6	12.2	7.82E-007
2	chr1p 13.00-33.00	BCAS2	breast carcinoma amplified sequence 2	0.54	11.17	2.00E-004
2	chr1p 13.00-33.00	WDR77	WD repeat domain 77	0.56	11.1	1.00E-004
2	chr1p 13.00-33.00	STXBP3	syntaxin binding protein 3	0.44	9.61	1.10E-003
2	chr1p 13.00-33.00	TRIM33	tripartite motif-containing 33	0.41	9.31	2.39E-002
2	chr1p 13.00-33.00	GADD45A	growth arrest and DNA-damage-inducible, alpha	0.45	8.94	6.70E-003
2	chr1p 13.00-33.00	DCLRE1B	DNA cross-link repair 1B (PSO2 homolog, S. cerevisiae)	0.36	8.72	9.01E-002
2	chr1p 13.00-33.00	SSX2IP	synovial sarcoma, X breakpoint 2 interacting protein	0.42	8.67	1.27E-002
2	chr1p 13.00-33.00	RHOC	ras homolog gene family, member C	0.4	8.56	1.06E-002
2	chr1p 13.00-33.00	PTBP2	polypyrimidine tract binding protein 2	0.34	8.54	3.64E-002
2	chr1p 13.00-33.00	HBXIP	hepatitis B virus x interacting protein	0.4	8.47	8.00E-003
2	chr1p 13.00-33.00	RBM15	RNA binding motif protein 15	0.33	8.44	2.43E-002
2	chr1p 13.00-33.00	CSDE1	cold shock domain containing E1, RNA-binding	0.59	8.15	6.00E-004
2	chr1p 13.00-33.00	GLMN	glomulin, FKBP associated protein	0.31	8.09	3.29E-002
2	chr1p 13.00-33.00	BCL10	B-cell CLL/lymphoma 10	0.29	8.05	4.26E-002
2	chr1p 13.00-33.00	RPL5	ribosomal protein L5	0.27	7.93	1.09E-001
2	chr1p 13.00-33.00	TRIM45	tripartite motif-containing 45	0.23	7.47	2.75E-002
2	chr1p 13.00-33.00	NOTCH2	Notch homolog 2 (Drosophila)	0.47	6.2	8.50E-003
2	chr1p 13.00-33.00	F3	coagulation factor III (thromboplastin, tissue factor)	0.25	3.47	2.21E-002
2	chr1p 13.00-33.00	LMO4	LIM domain only 4	0.21	3.44	9.22E-002

2	chr1p 13.00-33.00	DPYD	dihydropyrimidine dehydrogenase	0.18	3.33	3.52E-002
4	chr1q 23.00-43.00	NENF	neuron derived neurotrophic factor	0.52	10.65	3.94E-006
4	chr1q 23.00-43.00	KIF14	kinesin family member 14	0.44	9.58	2.10E-003
4	chr1q 23.00-43.00	NEK2	NIMA (never in mitosis gene a)-related kinase 2	0.49	9.48	1.33E-005
4	chr1q 23.00-43.00	IER5	immediate early response 5	0.41	9.21	1.70E-003
4	chr1q 23.00-43.00	SMYD2	SET and MYND domain containing 2	0.34	8.5	4.50E-003
4	chr1q 23.00-43.00	TIPRL	TIP41, TOR signaling pathway regulator-like (S. cerevisiae)	0.33	8.33	4.10E-003
4	chr1q 23.00-43.00	PARP1	poly (ADP-ribose) polymerase family, member 1	0.34	8.32	9.44E-002
4	chr1q 23.00-43.00	IQWD1	IQ motif and WD repeats 1	0.25	8.21	2.61E-002
4	chr1q 23.00-43.00	ARID4B	AT rich interactive domain 4B (RBP1-like)	0.3	8.1	5.10E-003
4	chr1q 23.00-43.00	LGALS8	lectin, galactoside-binding, soluble, 8 (galectin 8)	0.29	8.09	4.70E-002
4	chr1q 23.00-43.00	ASPM	asp (abnormal spindle) homolog, microcephaly associated (Drosophila)	0.29	8.03	4.37E-002
4	chr1q 23.00-43.00	H3F3A	H3 histone, family 3A	0.25	7.42	6.51E-002
4	chr1q 23.00-43.00	SMYD3	SET and MYND domain containing 3	0.46	7.22	1.75E-002
4	chr1q 23.00-43.00	STX6	syntaxin 6	0.15	7.03	2.13E-002
4	chr1q 23.00-43.00	TPR	translocated promoter region (to activated MET oncogene)	0.24	6.86	7.10E-003
4	chr1q 23.00-43.00	GUK1	guanylate kinase 1	0.32	6.57	9.71E-002
4	chr1q 23.00-43.00	PSEN2	presenilin 2 (Alzheimer disease 4)	0.54	6.16	4.47E-004
5	chr2p 12.00-24.10	COX7A2L	cytochrome c oxidase subunit VIIa polypeptide 2 like	0.65	12.64	4.95E-007
5	chr2p 12.00-24.10	THADA	thyroid adenoma associated	0.5	10.52	1.10E-003
5	chr2p 12.00-24.10	MSH6	mutS homolog 6 (E. coli)	0.48	9.76	1.40E-002
5	chr2p 12.00-24.10	MAP4K3	mitogen-activated protein kinase kinase kinase kinase 3	0.37	8.81	6.80E-003
5	chr2p 12.00-24.10	RHOQ	ras homolog gene family, member Q	0.35	8.6	2.00E-004
5	chr2p 12.00-24.10	VPS24	vacuolar protein sorting 24 homolog (S. cerevisiae)	0.26	7.5	7.72E-002
5	chr2p 12.00-24.10	DUSP11	dual specificity phosphatase 11 (RNA/RNP complex 1-interacting)	0.19	7.37	8.08E-002
5	chr2p 12.00-24.10	TP53I3	tumor protein p53 inducible protein 3	0.17	7.22	1.02E-001

5	chr2p 12.00-24.10	MTHFD2	methylenetetrahydrofolate dehydrogenase (NADP+ dependent) 2, methenyltetrahydrofolate cyclohydrolase	0.25	4.22	1.16E-001
5	chr2p 12.00-24.10	ITSN2	intersectin 2	0.21	3.76	6.92E-002
6	chr2q 23.00-24.00	ATF2	activating transcription factor 2	0.28	7.72	1.84E-002
6	chr2q 23.00-24.00	SLC20A1	solute carrier family 20 (phosphate transporter), member 1	0.35	7.23	3.45E-002
6	chr2q 23.00-24.00	NFE2L2	nuclear factor (erythroid-derived 2)-like 2	0.4	7.15	7.32E-002
7	chr3p 24.20-25.00	RAF1	v-raf-1 murine leukemia viral oncogene homolog 1	0.59	12.17	8.07E-007
7	chr3p 24.20-25.00	MKRN2	makorin, ring finger protein, 2	0.45	9.58	8.10E-002
7	chr3p 24.20-25.00	IQSEC1	IQ motif and Sec7 domain 1	0.57	8.82	2.66E-005
7	chr3p 24.20-25.00	PDCD6IP	programmed cell death 6 interacting protein	0.49	7.85	6.20E-003
8	chr3p 14.30-21.30	SACM1L	SAC1 suppressor of actin mutations 1-like (yeast)	0.64	13.43	2.18E-007
8	chr3p 14.30-21.30	BAP1	BRCA1 associated protein-1 (ubiquitin carboxy-terminal hydrolase)	0.59	12.05	9.14E-007
8	chr3p 14.30-21.30	RBM5	RNA binding motif protein 5	0.6	12	9.65E-007
8	chr3p 14.30-21.30	GNL3	guanine nucleotide binding protein-like 3 (nucleolar)	0.59	10.47	6.00E-004
8	chr3p 14.30-21.30	RBM6	RNA binding motif protein 6	0.5	10.46	2.10E-003
8	chr3p 14.30-21.30	USP4	ubiquitin specific peptidase 4 (proto-oncogene)	0.52	10.45	1.00E-004
8	chr3p 14.30-21.30	ENDOGL1	endonuclease G-like 1	0.42	9.45	3.30E-003
8	chr3p 14.30-21.30	CDC25A	cell division cycle 25 homolog A (S. pombe)	0.44	9.14	2.10E-003
8	chr3p 14.30-21.30	MAPKAPK3	mitogen-activated protein kinase-activated protein kinase 3	0.38	8.99	1.74E-002
8	chr3p 14.30-21.30	TUSC4	tumor suppressor candidate 4	0.35	8.7	5.42E-002
8	chr3p 14.30-21.30	UBE1L	ubiquitin-like modifier activating enzyme 7	0.21	7.91	4.92E-002
8	chr3p 14.30-21.30	ARIH2	ariadne homolog 2 (Drosophila)	0.46	7.36	9.00E-004
8	chr3p 14.30-21.30	MLH1	mutL homolog 1, colon cancer, nonpolyposis type 2 (E. coli)	0.28	6.1	8.50E-003
9	chr3p 13.00-14.20	CBLB	Cas-Br-M (murine) ecotropic retroviral transforming sequence b	0.39	8.95	6.23E-002

9	chr3p 13.00-14.20	MITF	microphthalmia-associated transcription factor	0.43	8.58	1.16E-001
10	chr3q 13.30-28.00	PIK3CA	phosphoinositide-3-kinase, catalytic, alpha polypeptide	0.34	11.35	3.40E-003
10	chr3q 13.30-28.00	RAB7	RAB7A, member RAS oncogene family	0.52	10.27	1.00E-003
10	chr3q 13.30-28.00	ZNF148	zinc finger protein 148	0.56	10.04	3.00E-004
10	chr3q 13.30-28.00	DNAJB11	DnaJ (Hsp40) homolog, subfamily B, member 11	0.57	9.35	3.00E-004
10	chr3q 13.30-28.00	RYK	RYK receptor-like tyrosine kinase	0.38	8.8	1.47E-002
10	chr3q 13.30-28.00	GSK3B	glycogen synthase kinase 3 beta	0.4	8.65	8.80E-003
10	chr3q 13.30-28.00	MRPL3	mitochondrial ribosomal protein L3	0.39	8.65	7.50E-003
10	chr3q 13.30-28.00	PIK3CB	phosphoinositide-3-kinase, catalytic, beta polypeptide	0.3	8.51	6.50E-003
10	chr3q 13.30-28.00	XRN1	5'-3' exoribonuclease 1	0.35	8.5	8.37E-002
10	chr3q 13.30-28.00	MLF1	myeloid leukemia factor 1	0.38	8.5	1.27E-002
10	chr3q 13.30-28.00	ZIC1	Zic family member 1 (odd-paired homolog, Drosophila)	0.37	8.33	1.50E-003
10	chr3q 13.30-28.00	GMPS	guanine monphosphate synthetase	0.28	7.91	3.41E-002
10	chr3q 13.30-28.00	PPP2R3A	protein phosphatase 2 (formerly 2A), regulatory subunit B'', alpha	0.35	7.9	4.10E-003
10	chr3q 13.30-28.00	DVL3	dishevelled, dsh homolog 3 (Drosophila)	0.37	7.88	1.12E-002
10	chr3q 13.30-28.00	GOLGB1	golgin B1, golgi integral membrane protein	0.28	7.67	2.46E-002
10	chr3q 13.30-28.00	IQCB1	IQ motif containing B1	0.15	7	7.99E-002
10	chr3q 13.30-28.00	MCM2	minichromosome maintenance complex component 2	0.09	6.38	3.66E-002
11	chr4p 16.30-16.30	TACC3	transforming, acidic coiled-coil containing protein 3	0.63	12.74	4.49E-007
11	chr4p 16.30-16.30	CTBP1	C-terminal binding protein 1	0.52	10.28	1.70E-003
11	chr4p 16.30-16.30	CHIC2	cysteine-rich hydrophobic domain 2	0.32	8.25	7.13E-002
14	chr5q 35.10-35.10	FBXW11	F-box and WD repeat domain containing 11	0.44	8.93	8.70E-003
14	chr5q 35.10-35.10	MAPK9	mitogen-activated protein kinase 9	0.38	8.82	5.00E-004
14	chr5q 35.10-35.10	BNIP1	BCL2/adenovirus E1B 19kDa interacting protein 1	0.3	8.23	4.41E-002
14	chr5q 35.10-35.10	PDLIM7	PDZ and LIM domain 7 (enigma)	0.32	5.46	3.40E-003
14	chr5q 35.10-35.10	NPM1	nucleophosmin (nucleolar phosphoprotein B23, numatrin)	0.37	4.85	1.80E-002

15	chr5q 31.00-31.10	PPP2CA	protein phosphatase 2 (formerly 2A), catalytic subunit, alpha isoform	0.43	9.43	1.60E-003
15	chr5q 31.00-31.10	CNOT8	CCR4-NOT transcription complex, subunit 8	0.44	9.33	5.70E-003
15	chr5q 31.00-31.10	RAD50	RAD50 homolog (S. cerevisiae)	0.39	9	3.60E-003
15	chr5q 31.00-31.10	KIF20A	kinesin family member 20A	0.31	8.87	1.56E-002
15	chr5q 31.00-31.10	EIF4EBP3	eukaryotic translation initiation factor 4E binding protein 3	0.39	8.57	1.84E-002
15	chr5q 31.00-31.10	APC	adenomatous polyposis coli	0.37	8.39	8.03E-002
15	chr5q 31.00-31.10	PHF15	PHD finger protein 15	0.22	8.13	5.00E-004
15	chr5q 31.00-31.10	HDAC3	histone deacetylase 3	0.37	7.79	3.97E-002
15	chr5q 31.00-31.10	H2AFY	H2A histone family, member Y	0.43	7.65	1.27E-002
15	chr5q 31.00-31.10	CDC23	cell division cycle 23 homolog (S. cerevisiae)	0.35	7.15	1.99E-002
15	chr5q 31.00-31.10	HSD17B4	hydroxysteroid (17-beta) dehydrogenase 4	0.35	6.68	3.17E-002
16	chr6p 21.20-21.30	ABCF1	ATP-binding cassette, sub-family F (GCN20), member 1	0.65	12.12	8.50E-007
16	chr6p 21.20-21.30	E2F3	E2F transcription factor 3	0.54	11.56	1.00E-004
16	chr6p 21.20-21.30	FANCE	Fanconi anemia, complementation group E	0.55	11.43	1.61E-002
16	chr6p 21.20-21.30	DEK	DEK oncogene (DNA binding)	0.51	10.85	3.00E-004
16	chr6p 21.20-21.30	TUBB	tubulin, beta	0.59	10.04	7.42E-006
16	chr6p 21.20-21.30	TTK	TTK protein kinase	0.44	9.73	1.00E-004
16	chr6p 21.20-21.30	RIPK1	receptor (TNFRSF)-interacting serine-threonine kinase 1	0.4	8.27	4.50E-003
16	chr6p 21.20-21.30	PBX2	pre-B-cell leukemia homeobox 2	0.31	8.26	4.61E-002
16	chr6p 21.20-21.30	BRD2	bromodomain containing 2	0.48	8.19	1.27E-002
16	chr6p 21.20-21.30	NOL7	nucleolar protein 7, 27kDa	0.63	7.56	4.40E-003
16	chr6p 21.20-21.30	PPP1R10	protein phosphatase 1, regulatory (inhibitor) subunit 10	0.22	7.33	2.37E-002
16	chr6p 21.20-21.30	BAK1	BCL2-antagonist/killer 1	0.33	6.74	1.20E-001
16	chr6p 21.20-21.30	HLA-DRB1	major histocompatibility complex, class II, DR beta 1	0.05	6.05	1.04E-001
16	chr6p 21.20-21.30	IRF4	interferon regulatory factor 4	0.28	5.63	1.11E-001
16	chr6p 21.20-21.30	VEGF	vascular endothelial growth factor A	0.27	4.49	3.23E-002
17	chr6q 21.00-27.00	AHI1	Abelson helper integration site 1	0.47	10.3	1.49E-002

17	chr6q 21.00-27.00	MYB	v-myb myeloblastosis viral oncogene homolog (avian)	0.34	9.8	1.78E-002
17	chr6q 21.00-27.00	PDCD2	programmed cell death 2	0.46	9.62	4.72E-002
17	chr6q 21.00-27.00	ECHDC1	enoyl Coenzyme A hydratase domain containing 1	0.46	9.22	9.30E-003
17	chr6q 21.00-27.00	GOPC	golgi associated PDZ and coiled-coil motif containing	0.5	8.97	1.11E-002
17	chr6q 21.00-27.00	MAP3K5	mitogen-activated protein kinase kinase kinase 5	0.17	8.92	4.95E-002
17	chr6q 21.00-27.00	HDAC2	histone deacetylase 2	0.38	8.92	3.90E-002
17	chr6q 21.00-27.00	AMD1	adenosylmethionine decarboxylase 1	0.31	8.82	8.59E-002
17	chr6q 21.00-27.00	FBXO5	F-box protein 5	0.4	8.73	3.72E-002
17	chr6q 21.00-27.00	CCNC	cyclin C	0.39	7.85	2.52E-002
17	chr6q 21.00-27.00	MAP3K7	mitogen-activated protein kinase kinase kinase 7	0.37	6.79	1.08E-001
18	chr7q 21.00-22.00	KRIT1	KRIT1, ankyrin repeat containing	0.56	11.53	1.57E-006
18	chr7q 21.00-22.00	DBF4	DBF4 homolog (S. cerevisiae)	0.47	10.08	2.60E-003
18	chr7q 21.00-22.00	AKAP9	A kinase (PRKA) anchor protein (yotiao) 9	0.41	9.23	4.50E-003
18	chr7q 21.00-22.00	DMTF1	cyclin D binding myb-like transcription factor 1	0.37	8.83	3.42E-002
18	chr7q 21.00-22.00	SRI	sorcin	0.46	8.2	9.60E-003
19	chr7q 21.00-31.00	ING3	inhibitor of growth family, member 3	0.48	9.31	7.40E-003
19	chr7q 21.00-31.00	POLR2J	polymerase (RNA) II (DNA directed) polypeptide J, 13.3kDa	0.38	8.61	6.91E-002
19	chr7q 21.00-31.00	RINT1	RAD50 interactor 1	0.41	8.38	4.68E-002
20	chr8p 11.00-22.00	LSM1	LSM1 homolog, U6 small nuclear RNA associated (S. cerevisiae)	0.57	10.88	3.00E-004
20	chr8p 11.00-22.00	MYST3	MYST histone acetyltransferase (monocytic leukemia) 3	0.44	9.23	3.01E-002
20	chr8p 11.00-22.00	FGFR1	fibroblast growth factor receptor 1 (fms-related tyrosine kinase 2, Pfeiffer syndrome)	0.37	9.12	1.95E-005
20	chr8p 11.00-22.00	PCM1	pericentriolar material 1	0.44	8.95	6.20E-002
20	chr8p 11.00-22.00	XPO7	exportin 7	0.5	8.41	5.50E-003
20	chr8p 11.00-22.00	RHOBTB2	Rho-related BTB domain containing 2	0.34	8.37	5.78E-002
20	chr8p 11.00-22.00	GTF2E2	general transcription factor IIE, polypeptide 2, beta 34kDa	0.37	8.28	1.09E-001

20	chr8p 11.00-22.00	EIF4EBP1	eukaryotic translation initiation factor 4E binding protein 1	0.22	8.12	6.07E-002
20	chr8p 11.00-22.00	WRN	Werner syndrome	0.49	7.09	1.82E-002
20	chr8p 11.00-22.00	VDAC3	voltage-dependent anion channel 3	0.53	4.31	4.60E-003
20	chr8p 11.00-22.00	TACC1	transforming, acidic coiled-coil containing protein 1	0.37	4.04	4.08E-002
21	chr8q 11.00-13.00	TCEA1	transcription elongation factor A (SII), 1	0.69	15.16	3.67E-008
21	chr8q 11.00-13.00	MCM4	minichromosome maintenance complex component 4	0.5	10.39	4.50E-003
21	chr8q 11.00-13.00	PABPC1	poly(A) binding protein, cytoplasmic 1	0.51	10.13	1.00E-004
21	chr8q 11.00-13.00	CHCHD7	coiled-coil-helix-coiled-coil-helix domain containing 7	0.38	8.98	2.39E-002
21	chr8q 11.00-13.00	EIF2C2	eukaryotic translation initiation factor 2C, 2	0.35	8.52	9.10E-003
21	chr8q 11.00-13.00	GGH	gamma-glutamyl hydrolase (conjugase, folylpolygammaglutamyl hydrolase)	0.31	8.29	6.48E-002
21	chr8q 11.00-13.00	PRKDC	protein kinase, DNA-activated, catalytic polypeptide	0.52	8.17	2.00E-003
21	chr8q 11.00-13.00	PTK2	PTK2 protein tyrosine kinase 2	0.32	7.87	9.20E-003
21	chr8q 11.00-13.00	TERF1	telomeric repeat binding factor (NIMA-interacting) 1	0.45	7.66	9.50E-003
21	chr8q 11.00-13.00	MTDH	metadherin	0.36	6.67	4.82E-002
21	chr8q 11.00-13.00	RB1CC1	RB1-inducible coiled-coil 1	0.48	6.59	1.66E-002
21	chr8q 11.00-13.00	OTUD6B	OTU domain containing 6B	0.25	6.5	5.19E-002
21	chr8q 11.00-13.00	EDD1	ubiquitin protein ligase E3 component n-recognin 5	0.4	6.44	1.98E-002
21	chr8q 11.00-13.00	ASPH	aspartate beta-hydroxylase	0.35	3.55	8.00E-003
22	chr9q 22.30-22.30	FANCC	Fanconi anemia, complementation group C	0.42	8.91	2.42E-002
22	chr9q 22.30-22.30	BAG1	BCL2-associated athanogene	0.37	8.75	2.80E-003
22	chr9q 22.30-22.30	TJP2	tight junction protein 2 (zona occludens 2)	0.4	7.3	7.40E-003
23	chr9q 34.00-34.10	SET	SET translocation (myeloid leukemia-associated)	0.66	13.63	1.77E-007
23	chr9q 34.00-34.10	COBRA1	cofactor of BRCA1	0.55	11.18	2.27E-006
23	chr9q 34.00-34.10	TSC1	tuberous sclerosis 1	0.5	10.45	1.10E-003
23	chr9q 34.00-34.10	RPL7A	ribosomal protein L7a	0.48	10.09	1.00E-004
23	chr9q 34.00-34.10	FBXW2	F-box and WD repeat domain containing 2	0.47	10	1.60E-003

23	chr9q 34.00-34.10	FPGS	folylpolyglutamate synthase	0.39	7.51	2.41E-002
23	chr9q 34.00-34.10	FNBP1	formin binding protein 1	0.22	6.7	4.17E-002
23	chr9q 34.00-34.10	RPL12	ribosomal protein L12	0.12	6.56	8.26E-002
23	chr9q 34.00-34.10	PPP2R4	protein phosphatase 2A activator, regulatory subunit 4	0.21	6.22	3.27E-002
24	chr10q 11.20-26.00	NFKB2	nuclear factor of kappa light polypeptide gene enhancer in B-cells 2 (p49/p100)	0.41	12.32	1.20E-003
24	chr10q 11.20-26.00	CDC2	cell division cycle 2, G1 to S and G2 to M	0.47	10.21	4.50E-003
24	chr10q 11.20-26.00	TIAL1	TIA1 cytotoxic granule-associated RNA binding protein-like 1	0.54	10.06	1.00E-004
24	chr10q 11.20-26.00	BCCIP	BRCA2 and CDKN1A interacting protein	0.5	9.94	8.23E-006
24	chr10q 11.20-26.00	ABLIM1	actin binding LIM protein 1	0.41	9.3	2.09E-002
24	chr10q 11.20-26.00	PRDX3	peroxiredoxin 3	0.56	9.11	1.98E-005
24	chr10q 11.20-26.00	TACC2	transforming, acidic coiled-coil containing protein 2	0.39	9.05	1.68E-002
24	chr10q 11.20-26.00	BMS1	BMS1 homolog, ribosome assembly protein (yeast)	0.34	8.76	1.00E-002
24	chr10q 11.20-26.00	MYST4	MYST histone acetyltransferase (monocytic leukemia) 4	0.34	8.53	1.14E-001
24	chr10q 11.20-26.00	SIRT1	sirtuin (silent mating type information regulation 2 homolog) 1 (S. cerevisiae)	0.34	8.44	3.22E-002
24	chr10q 11.20-26.00	PLCE1	phospholipase C, epsilon 1	0.3	8.16	4.15E-002
24	chr10q 11.20-26.00	WAPAL	wings apart-like homolog (Drosophila)	0.26	8.15	2.34E-002
24	chr10q 11.20-26.00	DDX50	DEAD (Asp-Glu-Ala-Asp) box polypeptide 50	0.3	8.1	7.20E-003
24	chr10q 11.20-26.00	ARL3	ADP-ribosylation factor-like 3	0.37	7.86	1.24E-002
24	chr10q 11.20-26.00	TRIM8	tripartite motif-containing 8	0.25	7.76	3.90E-003
24	chr10q 11.20-26.00	CASP7	caspase 7, apoptosis-related cysteine peptidase	0.35	7.61	2.79E-002
24	chr10q 11.20-26.00	PTEN	phosphatase and tensin homolog (mutated in multiple advanced cancers 1)	0.22	7.55	9.71E-002
24	chr10q 11.20-26.00	ANXA8	annexin A8	0.19	7.28	1.14E-001
24	chr10q 11.20-26.00	BMPR1A	bone morphogenetic protein receptor, type IA	0.43	6.44	8.80E-003
24	chr10q 11.20-26.00	TCF7L2	transcription factor 7-like 2 (T-cell specific, HMG-box)	0.34	6.21	7.18E-002

24	chr10q 11.20-26.00	UNC5B	unc-5 homolog B (C. elegans)	0.19	6.18	1.15E-001
24	chr10q 11.20-26.00	NCOA4	nuclear receptor coactivator 4	0.47	5.99	1.40E-003
24	chr10q 11.20-26.00	ANXA7	annexin A7	0.44	5.52	4.75E-002
24	chr10q 11.20-26.00	RAB11FIP2	RAB11 family interacting protein 2 (class I)	0.3	5.16	3.68E-002
24	chr10q 11.20-26.00	VDAC2	voltage-dependent anion channel 2	0.26	4.18	7.26E-002
24	chr10q 11.20-26.00	ANXA11	annexin A11	0.38	3.44	6.45E-002
25	chr11p 15.40-15.50	NUP98	nucleoporin 98kDa	0.48	10.26	6.00E-004
25	chr11p 15.40-15.50	SIGIRR	single immunoglobulin and toll-interleukin 1 receptor (TIR) domain	0.41	9.42	1.07E-002
25	chr11p 15.40-15.50	HRAS	v-Ha-ras Harvey rat sarcoma viral oncogene homolog	0.37	9.09	8.70E-003
25	chr11p 15.40-15.50	LRDD	leucine-rich repeats and death domain containing	0.39	8.84	1.08E-002
25	chr11p 15.40-15.50	RASSF7	Ras association (RalGDS/AF-6) domain family (N-terminal) member 7	0.28	8.8	3.09E-002
25	chr11p 15.40-15.50	STIM1	stromal interaction molecule 1	0.47	8.78	6.60E-003
25	chr11p 15.40-15.50	NAP1L4	nucleosome assembly protein 1-like 4	0.48	7.74	1.07E-002
25	chr11p 15.40-15.50	RRM1	ribonucleotide reductase M1	0.33	5.91	1.89E-002
26	chr11p 13.00-15.50	EIF4G2	eukaryotic translation initiation factor 4 gamma, 2	0.51	9.92	3.40E-003
26	chr11p 13.00-15.50	GPIAP1	cell cycle associated protein 1	0.37	8.64	4.80E-002
26	chr11p 13.00-15.50	TSG101	tumor susceptibility gene 101	0.49	7.96	6.40E-003
26	chr11p 13.00-15.50	RRAS2	related RAS viral (r-ras) oncogene homolog 2	0.26	7.58	1.76E-002
26	chr11p 13.00-15.50	SH2BP1	Ctr9, Paf1/RNA polymerase II complex component, homolog (S. cerevisiae)	0.3	7.55	1.08E-001
26	chr11p 13.00-15.50	CAT	catalase	0.14	7.05	6.82E-002
28	chr11q 21.00-23.20	MRE11A	MRE11 meiotic recombination 11 homolog A (S. cerevisiae)	0.51	12.72	4.55E-007
28	chr11q 21.00-23.20	ARCN1	archain 1	0.56	11.07	1.00E-004
28	chr11q 21.00-23.20	TMEM123	transmembrane protein 123	0.57	10.94	1.48E-002
28	chr11q 21.00-23.20	DDX10	DEAD (Asp-Glu-Ala-Asp) box polypeptide 10	0.49	9.8	1.00E-004
28	chr11q 21.00-23.20	RNF26	ring finger protein 26	0.51	9.01	2.20E-003
28	chr11q 21.00-23.20	C1QTNF5	C1q and tumor necrosis factor related protein 5	0.4	8.28	8.80E-003

28	chr11q 21.00-23.20	ARHGEF12	Rho guanine nucleotide exchange factor (GEF) 12	0.38	8.09	7.30E-002
28	chr11q 21.00-23.20	MMP1	matrix metallopeptidase 1 (interstitial collagenase)	0.04	3.89	6.61E-002
28	chr11q 21.00-23.20	BIRC2	baculoviral IAP repeat-containing 2	0.44	3.22	5.13E-002
29	chr11q 13.00-14.00	CHCHD8	coiled-coil-helix-coiled-coil-helix domain containing 8	0.51	10.71	1.30E-002
29	chr11q 13.00-14.00	PICALM	phosphatidylinositol binding clathrin assembly protein	0.23	3.4	3.11E-002
30	chr11q 13.00-13.00	CCND1	cyclin D1	0.46	8.99	1.74E-002
30	chr11q 13.00-13.00	FOLR1	folate receptor 1 (adult)	0.26	7.85	3.80E-002
31	chr12q 13.00-13.30	LEMD3	LEM domain containing 3	0.48	9.78	9.30E-003
31	chr12q 13.00-13.30	MCRS1	microspherule protein 1	0.33	9.05	2.40E-003
31	chr12q 13.00-13.30	STAT6	signal transducer and activator of transcription 6, interleukin-4 induced	0.34	8.39	2.47E-002
31	chr12q 13.00-13.30	ACVR1B	activin A receptor, type IB	0.37	8.36	7.60E-003
31	chr12q 13.00-13.30	JARID1A	jumonji, AT rich interactive domain 1A	0.31	8.33	4.25E-002
31	chr12q 13.00-13.30	STYK1	serine/threonine/tyrosine kinase 1	0.3	8.15	2.51E-002
31	chr12q 13.00-13.30	MAP3K12	mitogen-activated protein kinase kinase kinase 12	0.21	7.16	8.46E-002
32	chr12q 14.30-24.30	RPL6	ribosomal protein L6	0.64	12.73	7.00E-004
32	chr12q 14.30-24.30	MDM2	Mdm2, transformed 3T3 cell double minute 2, p53 binding protein (mouse)	0.35	11.52	3.10E-003
32	chr12q 14.30-24.30	DDX54	DEAD (Asp-Glu-Ala-Asp) box polypeptide 54	0.53	10.84	3.50E-003
32	chr12q 14.30-24.30	SART3	squamous cell carcinoma antigen recognized by T cells 3	0.55	9.84	3.50E-003
32	chr12q 14.30-24.30	PTPN11	protein tyrosine phosphatase, non-receptor type 11 (Noonan syndrome 1)	0.43	9.42	6.00E-004
32	chr12q 14.30-24.30	RAN	RAN, member RAS oncogene family	0.38	8.75	3.89E-002
32	chr12q 14.30-24.30	PEBP1	phosphatidylethanolamine binding protein 1	0.26	8.38	1.52E-002
32	chr12q 14.30-24.30	ATP2A2	ATPase, Ca++ transporting, cardiac muscle, slow twitch 2	0.46	7.36	7.40E-003
32	chr12q 14.30-24.30	PXN	paxillin	0.33	6.32	4.50E-002
32	chr12q 14.30-24.30	TXNRD1	thioredoxin reductase 1	0.26	5.48	7.20E-002
32	chr12q 14.30-24.30	RNF34	ring finger protein 34	0.32	4.76	6.38E-002

33	chr13q 12.30-21.20	ERCC5	excision repair cross-complementing rodent repair deficiency, complementation group 5 (xeroderma pigmentosum, complementation group G (Cockayne syndrome))	0.54	20.08	4.00E-004
33	chr13q 12.30-21.20	TPT1	tumor protein, translationally-controlled 1	0.66	13.89	1.00E-004
33	chr13q 12.30-21.20	EFNB2	ephrin-B2	0.19	12.44	3.36E-002
33	chr13q 12.30-21.20	GTF3A	general transcription factor IIIA	0.54	9.91	4.00E-004
33	chr13q 12.30-21.20	BRCA2	breast cancer 2, early onset	0.46	8.94	3.56E-002
33	chr13q 12.30-21.20	HMGB1	high-mobility group box 1	0.37	8.64	1.00E-002
33	chr13q 12.30-21.20	INTS6	integrator complex subunit 6	0.34	8.21	3.41E-002
33	chr13q 12.30-21.20	RAP2A	RAP2A, member of RAS oncogene family	0.28	7.71	4.97E-002
33	chr13q 12.30-21.20	CRYL1	crystallin, lambda 1	0.27	7.46	5.85E-002
34	chr14q 11.20-32.32	PNMA1	paraneoplastic antigen MA1	0.58	12.47	5.91E-007
34	chr14q 11.20-32.32	PPM1A	protein phosphatase 1A (formerly 2C), magnesium-dependent, alpha isoform	0.6	12.08	2.00E-004
34	chr14q 11.20-32.32	MNAT1	menage a trois homolog 1, cyclin H assembly factor (Xenopus laevis)	0.55	11.98	4.00E-004
34	chr14q 11.20-32.32	GARNL1	GTPase activating Rap/RanGAP domain-like 1	0.69	11.2	2.21E-006
34	chr14q 11.20-32.32	GMPR2	guanosine monophosphate reductase 2	0.51	10.65	9.00E-004
34	chr14q 11.20-32.32	GPHN	gephyrin	0.49	10.3	7.00E-004
34	chr14q 11.20-32.32	NUMB	numb homolog (Drosophila)	0.45	9.88	1.68E-002
34	chr14q 11.20-32.32	LTB4R	leukotriene B4 receptor	0.46	9.81	9.48E-006
34	chr14q 11.20-32.32	DHRS4	dehydrogenase/reductase (SDR family) member 4	0.44	9.62	1.15E-005
34	chr14q 11.20-32.32	ALKBH1	alkB, alkylation repair homolog 1 (E. coli)	0.42	9.45	5.56E-002
34	chr14q 11.20-32.32	MPP5	membrane protein, palmitoylated 5 (MAGUK p55 subfamily member 5)	0.44	9.4	7.50E-003
34	chr14q 11.20-32.32	SDCCAG1	serologically defined colon cancer antigen 1	0.41	9.32	4.41E-002
34	chr14q 11.20-32.32	XRCC3	X-ray repair complementing defective repair in Chinese hamster cells 3	0.38	8.91	8.72E-002
34	chr14q 11.20-32.32	TDP1	tyrosyl-DNA phosphodiesterase 1	0.36	8.88	1.60E-003
34	chr14q 11.20-32.32	PNN	pinin, desmosome associated protein	0.33	8.82	8.00E-004
34	chr14q 11.20-32.32	MAP3K9	mitogen-activated protein kinase kinase kinase 9	0.36	8.77	1.09E-002

34	chr14q 11.20-32.32	OTUB2	OTU domain, ubiquitin aldehyde binding 2	0.29	8.77	1.17E-002
34	chr14q 11.20-32.32	CALM1	calmodulin 1 (phosphorylase kinase, delta)	0.35	8.59	5.66E-002
34	chr14q 11.20-32.32	KLHDC2	kelch domain containing 2	0.34	8.55	2.60E-002
34	chr14q 11.20-32.32	NOVA1	neuro-oncological ventral antigen 1	0.3	8.46	2.11E-002
34	chr14q 11.20-32.32	BMP4	bone morphogenetic protein 4	0.28	8.25	1.43E-002
34	chr14q 11.20-32.32	MLH3	mutL homolog 3 (E. coli)	0.42	8.25	5.60E-003
34	chr14q 11.20-32.32	TRIP11	thyroid hormone receptor interactor 11	0.32	8.18	5.30E-002
34	chr14q 11.20-32.32	ARID4A	AT rich interactive domain 4A (RBP1-like)	0.43	8.14	2.81E-002
34	chr14q 11.20-32.32	WDR22	WD repeat domain 22	0.39	8.06	1.11E-002
34	chr14q 11.20-32.32	MAX	MYC associated factor X	0.29	7.69	2.57E-002
34	chr14q 11.20-32.32	GOLGA5	golgi autoantigen, golgin subfamily a, 5	0.39	7.15	2.10E-003
34	chr14q 11.20-32.32	HIF1A	hypoxia-inducible factor 1, alpha subunit (basic helix-loop-helix transcription factor)	0.29	6.93	1.50E-002
34	chr14q 11.20-32.32	FOS	v-fos FBJ murine osteosarcoma viral oncogene homolog	0.18	6.29	5.08E-002
34	chr14q 11.20-32.32	DYNC1H1	dynein, cytoplasmic 1, heavy chain 1	0.23	5.85	9.74E-002
35	chr15q 22.00-26.10	ANP32A	acidic (leucine-rich) nuclear phosphoprotein 32 family, member A	0.54	11.64	1.40E-006
35	chr15q 22.00-26.10	BLM	Bloom syndrome	0.46	11.04	2.61E-006
35	chr15q 22.00-26.10	ADPGK	ADP-dependent glucokinase	0.52	10.85	9.20E-003
35	chr15q 22.00-26.10	ISG20	interferon stimulated exonuclease gene 20kDa	0.51	10.67	1.70E-003
35	chr15q 22.00-26.10	PSMA4	proteasome (prosome, macropain) subunit, alpha type, 4	0.49	10.28	6.00E-004
35	chr15q 22.00-26.10	RAB11A	RAB11A, member RAS oncogene family	0.49	9.91	8.54E-006
35	chr15q 22.00-26.10	CSK	c-src tyrosine kinase	0.39	9.36	6.84E-002
35	chr15q 22.00-26.10	SNRPA1	small nuclear ribonucleoprotein polypeptide A'	0.44	9.36	6.29E-002
35	chr15q 22.00-26.10	GLCE	glucuronic acid epimerase	0.39	8.97	1.97E-002
35	chr15q 22.00-26.10	NARG2	NMDA receptor regulated 2	0.37	8.6	1.50E-003
35	chr15q 22.00-26.10	SIN3A	SIN3 homolog A, transcription regulator (yeast)	0.33	8.28	2.35E-002
35	chr15q 22.00-26.10	AKAP13	A kinase (PRKA) anchor protein 13	0.26	8.27	4.50E-003
35	chr15q 22.00-26.10	CPEB1	cytoplasmic polyadenylation element binding protein 1	0.27	7.67	1.06E-001

35	chr15q 22.00-26.10	BNIP2	BCL2/adenovirus E1B 19kDa interacting protein 2	0.25	6.88	9.08E-002
35	chr15q 22.00-26.10	PKM2	pyruvate kinase, muscle	0.28	6.83	7.50E-003
35	chr15q 22.00-26.10	ADAM10	ADAM metallopeptidase domain 10	0.34	5.31	2.07E-002
35	chr15q 22.00-26.10	MAP2K1	mitogen-activated protein kinase kinase 1	0.3	4.31	3.03E-002
36	chr16p 13.10-13.30	TELO2	TEL2, telomere maintenance 2, homolog (S. cerevisiae)	0.45	9.72	1.55E-002
36	chr16p 13.10-13.30	E4F1	E4F transcription factor 1	0.41	9.28	1.00E-003
36	chr16p 13.10-13.30	GSPT1	G1 to S phase transition 1	0.39	8.68	7.60E-003
36	chr16p 13.10-13.30	ABCC1	ATP-binding cassette, sub-family C (CFTR/MRP), member 1	0.34	8.54	3.70E-003
36	chr16p 13.10-13.30	MRPL28	mitochondrial ribosomal protein L28	0.28	8.02	4.51E-002
36	chr16p 13.10-13.30	MKL2	MKL/myocardin-like 2	0.25	7.66	1.44E-002
36	chr16p 13.10-13.30	CDR2	cerebellar degeneration-related protein 2, 62kDa	0.18	7.02	1.15E-001
36	chr16p 13.10-13.30	MPG	N-methylpurine-DNA glycosylase	0.22	4.15	9.63E-002
36	chr16p 13.10-13.30	SULT1A1	sulfotransferase family, cytosolic, 1A, phenol-preferring, member 1	0.26	3.8	3.89E-002
37	chr16q 22.10-24.30	SPG7	spastic paraplegia 7 (pure and complicated autosomal recessive)	0.45	11.17	1.77E-002
37	chr16q 22.10-24.30	PARD6A	par-6 partitioning defective 6 homolog alpha (C. elegans)	0.51	10.41	6.00E-004
37	chr16q 22.10-24.30	CYBA	cytochrome b-245, alpha polypeptide	0.4	9.02	5.00E-004
37	chr16q 22.10-24.30	TCF25	transcription factor 25 (basic helix-loop-helix)	0.37	8.74	5.67E-002
37	chr16q 22.10-24.30	CYLD	cylindromatosis (turban tumor syndrome)	0.37	8.58	2.22E-002
37	chr16q 22.10-24.30	FANCA	Fanconi anemia, complementation group A	0.36	8.57	1.67E-002
37	chr16q 22.10-24.30	ST3GAL2	ST3 beta-galactoside alpha-2,3-sialyltransferase 2	0.23	8.15	4.90E-003
37	chr16q 22.10-24.30	APRT	adenine phosphoribosyltransferase	0.3	7.88	4.43E-002
37	chr16q 22.10-24.30	SIAH1	seven in absentia homolog 1 (Drosophila)	0.23	7.61	9.60E-002
37	chr16q 22.10-24.30	TERF2IP	telomeric repeat binding factor 2, interacting protein	0.2	6.46	1.06E-001
37	chr16q 22.10-24.30	CDK10	cyclin-dependent kinase 10	0.19	3.31	1.91E-002
38	chr17p 13.00-13.30	FAM57A	family with sequence similarity 57, member A	0.51	10.76	6.00E-004

38	chr17p 13.00-13.30	DPH1	DPH1 homolog (S. cerevisiae)	0.48	10.3	6.00E-003
38	chr17p 13.00-13.30	ABR	active BCR-related gene	0.41	9.81	4.09E-002
38	chr17p 13.00-13.30	MYBBP1A	MYB binding protein (P160) 1a	0.44	9.67	4.00E-004
38	chr17p 13.00-13.30	GEMIN4	gem (nuclear organelle) associated protein 4	0.41	9.25	8.30E-003
38	chr17p 13.00-13.30	MYO1C	myosin IC	0.27	7.85	5.70E-003
38	chr17p 13.00-13.30	TP53	tumor protein p53	0.26	6.21	2.58E-002
38	chr17p 13.00-13.30	ITGAE	integrin, alpha E (antigen CD103, human mucosal lymphocyte antigen 1; alpha polypeptide)	0.56	4.74	2.08E-003
38	chr17p 13.00-13.30	AURKB	aurora kinase B	0.36	4.72	5.21E-002
38	chr17p 13.00-13.30	CRK	v-crk sarcoma virus CT10 oncogene homolog (avian)	0.48	3.28	1.05E-002
38	chr17p 13.00-13.30	DERL2	Der1-like domain family, member 2	0.54	3.09	1.29E-002
39	chr17q 11.20-22.00	PSMC3IP	PSMC3 interacting protein	0.59	11.04	2.62E-006
39	chr17q 11.20-22.00	CASC3	cancer susceptibility candidate 3	0.56	9.65	2.00E-003
39	chr17q 11.20-22.00	BRCA1	breast cancer 1, early onset	0.44	9.45	1.40E-002
39	chr17q 11.20-22.00	PPARBP	mediator complex subunit 1	0.49	9.16	5.00E-004
39	chr17q 11.20-22.00	SUZ12	suppressor of zeste 12 homolog (Drosophila)	0.42	9.03	5.50E-003
39	chr17q 11.20-22.00	BECN1	beclin 1, autophagy related	0.34	8.49	3.12E-002
39	chr17q 11.20-22.00	PIGS	phosphatidylinositol glycan anchor biosynthesis, class S	0.33	8.37	4.41E-002
39	chr17q 11.20-22.00	STAT3	signal transducer and activator of transcription 3 (acute-phase response factor)	0.24	8.24	3.82E-002
39	chr17q 11.20-22.00	NBR1	neighbor of BRCA1 gene 1	0.34	8.21	9.30E-003
39	chr17q 11.20-22.00	MLX	MAX-like protein X	0.37	8.2	2.94E-002
39	chr17q 11.20-22.00	POLDIP2	polymerase (DNA-directed), delta interacting protein 2	0.26	7.94	1.97E-002
39	chr17q 11.20-22.00	LASP1	LIM and SH3 protein 1	0.31	7.79	4.40E-003
39	chr17q 11.20-22.00	NLK	nemo-like kinase	0.25	7.48	1.30E-002
39	chr17q 11.20-22.00	TRAF4	TNF receptor-associated factor 4	0.18	7.34	6.76E-002
39	chr17q 11.20-22.00	TOP2A	topoisomerase (DNA) II alpha 170kDa	0.36	7.24	1.89E-002
39	chr17q 11.20-22.00	PCGF2	polycomb group ring finger 2	0.38	7.11	8.94E-002

39	chr17q 11.20-22.00	PIP5K2B	phosphatidylinositol-5-phosphate 4-kinase, type II, beta	0.3	5.13	4.03E-002
39	chr17q 11.20-22.00	BLMH	bleomycin hydrolase	0.34	4.9	1.96E-002
40	chr17q 22.00-23.20	GRB2	growth factor receptor-bound protein 2	0.56	9.83	2.40E-003
40	chr17q 22.00-23.20	MIRN21	microRNA 21	0.43	9.57	1.00E-004
40	chr17q 22.00-23.20	H3F3B	H3 histone, family 3B (H3.3B)	0.46	8.5	3.10E-003
40	chr17q 22.00-23.20	CLTC	clathrin, heavy chain (Hc)	0.45	8.33	1.57E-002
40	chr17q 22.00-23.20	RPS6KB1	ribosomal protein S6 kinase, 70kDa, polypeptide 1	0.57	8.23	5.00E-004
40	chr17q 22.00-23.20	PPM1D	protein phosphatase 1D magnesium-dependent, delta isoform	0.3	8.14	2.00E-002
40	chr17q 22.00-23.20	PRKCA	protein kinase C, alpha	0.39	7.58	1.65E-002
40	chr17q 22.00-23.20	MMD	monocyte to macrophage differentiation-associated	0.38	7.33	1.00E-002
41	chr18p 11.20-11.31	TYMS	thymidylate synthetase	0.44	8.01	7.70E-003
42	chr19q 12.00-13.40	SUPT5H	suppressor of Ty 5 homolog (S. cerevisiae)	0.52	10.81	6.20E-003
42	chr19q 12.00-13.40	PRMT1	protein arginine methyltransferase 1	0.4	9.61	2.30E-003
42	chr19q 12.00-13.40	RPS19	ribosomal protein S19	0.36	9.21	1.10E-003
42	chr19q 12.00-13.40	PSCD2	pleckstrin homology, Sec7 and coiled-coil domains 2 (cytohesin-2)	0.39	9.06	5.46E-002
42	chr19q 12.00-13.40	PDCD5	programmed cell death 5	0.38	8.97	1.60E-002
42	chr19q 12.00-13.40	SERTAD3	SERTA domain containing 3	0.37	8.67	2.38E-002
42	chr19q 12.00-13.40	POLD1	polymerase (DNA directed), delta 1, catalytic subunit 125kDa	0.33	8.42	8.81E-002
42	chr19q 12.00-13.40	ERF	Ets2 repressor factor	0.29	8.23	5.60E-003
42	chr19q 12.00-13.40	BAX	BCL2-associated X protein	0.19	8.21	3.84E-002
42	chr19q 12.00-13.40	XRCC1	X-ray repair complementing defective repair in Chinese hamster cells 1	0.37	8.16	7.33E-002
42	chr19q 12.00-13.40	KDELR1	KDEL (Lys-Asp-Glu-Leu) endoplasmic reticulum protein retention receptor 1	0.31	8.07	2.63E-002
42	chr19q 12.00-13.40	PVR	poliovirus receptor	0.29	8.06	2.48E-002
42	chr19q 12.00-13.40	GRLF1	glucocorticoid receptor DNA binding factor 1	0.3	7.97	1.02E-001
42	chr19q 12.00-13.40	ZNF224	zinc finger protein 224	0.23	7.52	6.47E-002
42	chr19q 12.00-13.40	RENT1	UPF1 regulator of nonsense transcripts homolog (yeast)	0.36	7.51	6.60E-003

42	chr19q 12.00-13.40	TFPT	TCF3 (E2A) fusion partner (in childhood Leukemia)	0.21	7.45	5.01E-002
42	chr19q 12.00-13.40	CARD8	caspase recruitment domain family, member 8	0.22	7.35	2.54E-002
42	chr19q 12.00-13.40	PPP1R13L	protein phosphatase 1, regulatory (inhibitor) subunit 13 like	0.17	7.21	1.50E-002
42	chr19q 12.00-13.40	ERCC2	excision repair cross-complementing rodent repair deficiency, complementation group 2 (xeroderma pigmentosum D)	0.29	7.03	2.75E-002
42	chr19q 12.00-13.40	BCL3	B-cell CLL/lymphoma 3	0.13	5.98	1.17E-001
42	chr19q 12.00-13.40	ACTN4	actinin, alpha 4	0.39	4.47	1.78E-002
43	chr20p 13.00-13.00	PTPRA	protein tyrosine phosphatase, receptor type, A	0.62	12.31	2.00E-004
45	chr20q 13.00-13.31	AURKA	aurora kinase A	0.38	7.36	9.33E-002
45	chr20q 13.00-13.31	CTSZ	cathepsin Z	0.22	6.91	9.40E-002
46	chr20q 11.20-13.20	NCOA3	nuclear receptor coactivator 3	0.22	8.47	7.11E-002
46	chr20q 11.20-13.20	CD40	CD40 molecule, TNF receptor superfamily member 5	0.33	7.02	5.73E-002
47	chr21q 22.10-22.30	SOD1	superoxide dismutase 1, soluble (amyotrophic lateral sclerosis 1 (adult))	0.38	8.98	4.96E-002
47	chr21q 22.10-22.30	SON	SON DNA binding protein	0.55	8.83	3.40E-003
47	chr21q 22.10-22.30	TIAM1	T-cell lymphoma invasion and metastasis 1	0.22	8.72	3.10E-002
47	chr21q 22.10-22.30	U2AF1	U2 small nuclear RNA auxiliary factor 1	0.49	7.28	1.20E-003
47	chr21q 22.10-22.30	IL10RB	interleukin 10 receptor, beta	0.19	6.32	9.68E-002
47	chr21q 22.10-22.30	PTTG1IP	pituitary tumor-transforming 1 interacting protein	0.21	3.75	1.22E-002
48	chr22q 12.20-13.10	TXN2	thioredoxin 2	0.55	11.26	2.08E-006
48	chr22q 12.20-13.10	GTPBP1	GTP binding protein 1	0.46	9.91	4.00E-004
48	chr22q 12.20-13.10	MAPK1	mitogen-activated protein kinase 1	0.43	9.43	1.70E-003
48	chr22q 12.20-13.10	PRR5	proline rich 5 (renal)	0.4	9.27	2.47E-002
48	chr22q 12.20-13.10	TOM1	target of myb1 (chicken)	0.44	8.62	2.34E-002
48	chr22q 12.20-13.10	AP1B1	adaptor-related protein complex 1, beta 1 subunit	0.29	7.99	5.94E-002
48	chr22q 12.20-13.10	ST13	suppression of tumorigenicity 13 (colon carcinoma) (Hsp70 interacting protein)	0.53	5.4	1.01E-003

48	chr22q 12.20-13.10	SCO2	SCO cytochrome oxidase deficient homolog 2 (yeast)	0.33	5.02	1.80E-003
48	chr22q 12.20-13.10	GGT1	gamma-glutamyltransferase 1	0.11	4.3	2.21E-002
49	chr23q 11.20-28.00	HPRT1	hypoxanthine phosphoribosyltransferase 1 (Lesch-Nyhan syndrome)	0.55	10.44	4.90E-006
49	chr23q 11.20-28.00	MCTS1	malignant T cell amplified sequence 1	0.49	9.33	2.00E-004
49	chr23q 11.20-28.00	IRAK1	interleukin-1 receptor-associated kinase 1	0.4	9.04	1.76E-002
49	chr23q 11.20-28.00	KIF4A	kinesin family member 4A	0.35	9.04	2.21E-002
49	chr23q 11.20-28.00	VBP1	von Hippel-Lindau binding protein 1	0.39	8.95	1.40E-003
49	chr23q 11.20-28.00	OTUD5	OTU domain containing 5	0.4	8.95	2.80E-003
49	chr23q 11.20-28.00	MTCP1	mature T-cell proliferation 1	0.35	8.29	1.01E-002
49	chr23q 11.20-28.00	SPANXC	SPANX family, member C	0.31	8.2	4.40E-003
49	chr23q 11.20-28.00	CTPS2	CTP synthase II	0.31	7.85	1.93E-002
49	chr23q 11.20-28.00	ARD1A	ARD1 homolog A, N-acetyltransferase (S. cerevisiae)	0.3	7.78	5.31E-002
49	chr23q 11.20-28.00	FMR1	fragile X mental retardation 1	0.24	7.57	1.08E-001
49	chr23q 11.20-28.00	ENOX2	ecto-NOX disulfide-thiol exchanger 2	0.25	7.53	2.84E-002
49	chr23q 11.20-28.00	UTP14A	UTP14, U3 small nucleolar ribonucleoprotein, homolog A (yeast)	0.26	7.52	1.00E-002
49	chr23q 11.20-28.00	AKAP14	A kinase (PRKA) anchor protein 14	0.42	7.48	1.35E-002
49	chr23q 11.20-28.00	BCAP31	B-cell receptor-associated protein 31	0.3	7.39	2.71E-002
49	chr23q 11.20-28.00	CETN2	centrin, EF-hand protein, 2	0.27	6.98	3.67E-002
49	chr23q 11.20-28.00	DKC1	dyskeratosis congenita 1, dyskerin	0.26	6.66	1.12E-001
49	chr23q 11.20-28.00	COL4A5	collagen, type IV, alpha 5 (Alport syndrome)	0.3	5.98	1.77E-002
49	chr23q 11.20-28.00	DLG3	discs, large homolog 3 (neuroendocrine-dlg, Drosophila)	0.24	5.8	8.41E-002

Summary of clusters of methylated genes

cluster index	methylated genes	# affected genes	# affected mirs
1	EGFR WT1 TCL1A FLT3 CHGA GABRB3 RET GAS7 SMO IGF2 PAX3 FLT4 FEV PAX7 HOXC13 ZIM2	490	11
2	BCR BCL7A	157	10
3	PAX8	83	0
4	PPARG	63	0
5	TCF1	61	2
6	SYK	37	0
7	CD38	34	0
8	LCK	17	0
9	MAFB	17	0
10	ZNF331	14	1
11	KIT	13	0
12	FLI1	12	0
13	IRF4	11	0
14	CBFA2T3	10	1
